# Global Trends in Incidence Rates of Primary Adult Liver Cancers: A Systematic Review and Meta-Analysis

**DOI:** 10.3389/fonc.2020.00171

**Published:** 2020-02-28

**Authors:** Paramita Dasgupta, Chloe Henshaw, Danny R. Youlden, Paul J. Clark, Joanne F. Aitken, Peter D. Baade

**Affiliations:** ^1^Cancer Research Centre, Cancer Council Queensland, Brisbane, QLD, Australia; ^2^Menzies Health Institute Queensland, Griffith University, Gold Coast, QLD, Australia; ^3^Faculty of Medicine, University of Queensland, Brisbane, QLD, Australia; ^4^Mater Research Institute, Brisbane, QLD, Australia; ^5^Princess Alexandra Hospital & Mater Hospital, Brisbane, QLD, Australia; ^6^Institute for Resilient Regions, University of Southern Queensland, Toowoomba, QLD, Australia; ^7^School of Public Health, University of Queensland, Brisbane, QLD, Australia; ^8^School of Mathematical Sciences, Queensland University of Technology, Brisbane, QLD, Australia

**Keywords:** liver cancer, incidence, trends, hepatocellular carcinoma, systematic review, meta-analysis

## Abstract

**Background:** Primary liver cancer is a leading cause of cancer deaths worldwide. Global burden varies, reflecting geographical distribution of viral hepatitis. Our objective was to perform a systematic review and meta-analysis of published current trends in incidence of adult liver cancers and histological types worldwide.

**Methods:** This study used systematic searches of PubMed, Embase, CINAHL, and Web of Science databases for English-language peer-reviewed articles published from 1 January 2008 to 01 September 2019. Inclusion criteria were population-based studies of adult liver cancer patients with quantitative estimates of temporal trends in incidence for liver cancers and/or histological types. For multiple studies from the same geographical area, only the publication that reported the most recent trends for the same cancer type and population subgroup was included. Review was conducted per PRISMA guidelines. Two authors independently extracted data and critically assessed studies. Proposed contributors to observed trends were extracted from included articles. Study-specific estimates of the annual percentage change (APC) in incidence rates with 95% confidence intervals (CIs) were pooled using random-effects meta-analysis models. Heterogeneity was measured using the *I*^2^ statistics and publication bias evaluated using funnel plots and Egger's tests.

**Results:** Overall, 53 studies met the inclusion criteria, of which 31 were included in the meta-analysis. Overall, pooled APC estimates were +0.8 (95% CI −0.3, +2.0) for liver cancers combined, +2.6 (95% CI +1.2, +4.0) for hepatocellular carcinoma (HCC), and +4.3 (95% CI +2.5, +6.1) for intrahepatic cholangiocarcinoma. Subgroup analyses indicated increasing trends for liver cancers (APC +3.2, 95% CI +2.5, +3.9) and HCC (APC +3.6, 95% CI +2.9, +4.4) in the region of North America/Europe/Australia, whereas corresponding trends were decreasing (APC −1.7, 95% CI −2.2, −1.1) and stable (APC −0.7, 95% CI −1.9, +0.5) in Asia, respectively.

**Conclusions:** Incidence is increasing for adult liver cancers and HCC in Western countries, whereas trends are decreasing in the Asian region, although still remaining high. Our findings highlight the importance of viral hepatitis control and lifestyle interventions to reduce global liver cancer burden. Ongoing surveillance is also vital to detect early shifts in incidence trends.

## Introduction

Primary liver cancer is the sixth most commonly diagnosed cancer and the fourth leading cause of cancer mortality worldwide, with an estimated 841,000 cases (9.3 cases per 100,000 person-years) and 782,000 deaths (8.5 deaths per 100,000 person-years) in 2018 (1). It is more common in men than in women (1). Outcomes are poor with an estimated 5-year net survival of 19% (2009–2015) (2) and an average 19 years of life lost per death in the USA (3).

The two major histological types are hepatocellular carcinoma (HCC), which comprises around 75% of all liver cancer cases, and intrahepatic cholangiocarcinoma (ICC, about 12–15%) (4). Liver cancer incidence rates vary from 5.1 per 100,000 person-years in Europe to 17.7 per 100,000 person-years in eastern Asia (5). This wide geographical variation primarily reflects regional differences in the prevalence of risk factors, especially for HCC (6). HCC generally occurs in the presence of liver disease or cirrhosis (7), and at least 60% of HCC cases worldwide are caused by viral hepatitis (8). Chronic hepatitis B virus (HBV), typically acquired at birth or early childhood, remains the leading cause in most high-risk HCC areas. In developed countries, the predominant etiology is more likely to be hepatitis C virus (HCV) infections acquired later in life (8). Other risk factors include dietary aflatoxin exposure, smoking, and high alcohol consumption, with nonalcoholic fatty liver disease (NAFLD) associated with obesity and diabetes increasingly emerging as a key contributor in the USA and other Western countries (6, 7).

Compared to HCC, relatively little is known about the etiology of ICC, especially in Western countries. Commonly cited risk factors include cirrhosis, viral hepatitis, primary sclerosing cholangitis, and liver fluke infections (in Asia) (9, 10).

Up-to-date accurate population-based data on cancer incidence and associated trends over time are fundamental for understanding the future cancer burden and informing health care planning and resource allocation. In 2014, an estimated 71% of all incident liver cancers diagnosed in the USA were attributable to potentially modifiable risk factors (11). A better understanding of the wide geographical variation in the global liver cancer burden, both within and between countries, may suggest novel targets for prevention strategies.

We performed a systematic review and meta-analysis to synthesize published evidence on contemporary trends in adult liver cancer incidence globally and to discuss the proposed contributors to the observed incidence trends. This review is intended to identify gaps in knowledge and contribute to the design of further etiological studies.

## Methods

### Definitions

Primary liver cancer included the International Classification of Diseases for Oncology, 3rd Edition (ICD-O-3), or International Classification of Disease, Tenth Edition (ICD-10), site code C22. Histological types were typically defined as HCC (ICD-O-3 site code C22.0, morphology codes M8170–M8175), ICC (ICD-O-3 site code C22.1, M8160), or combined hepatocellular cholangiocarcinoma (cHCC-CC, M8180).

Countries were grouped according to the 2019 United Nations Human Development Index (HDI) classification system (12) of developed (high/very high HDI) or developing (low/medium HDI).

### Clinical Question

This review was conducted according to published PRISMA guidelines (13). The clinical question to guide the review was clearly defined following the structured PICOS framework and agreed upon before commencing the review process.

The PICOS question is: Among adults diagnosed with invasive liver cancer (Participants), was there any effect of calendar time (Intervention) from the start of the study time period (Comparisons) in the incidence rates (Outcomes) using population-based cohorts from cancer registries (Study design)?

### Searches

The electronic databases PubMed, Embase, CINAHL, and Web of Science were systematically searched for all indexed articles from 1 January 2008 to focus on the most recent trends. Final searches were undertaken on 1 September 2019. Search strategies used selected subject headings and key words related to liver cancer, for example, “liver cancer,” “liver neoplasms,” “liver tumor,” “hepatocellular carcinoma,” “hepatic cancers,” “hepatic tumors,” and “intrahepatic cholangiocarcinoma,” combined with (“incidence” and “trends” and “epidemiology”) (Supplementary File 1). Reference lists of reviews and retrieved articles were checked to identify potentially relevant articles.

### Study Selection

Studies were eligible if they met the following predetermined selection criteria:

1) The study is population based.
2) The study cohort included adults aged at least 15 years diagnosed with liver cancer and/or subgroups.
3) Outcome measure was incidence rates.
4) Quantitative estimates of temporal trends in incidence rates were presented as percentage change over time.

The scope of the review was limited to English-language peer-reviewed original research articles. Studies that only reported overall numbers or proportions of cases by time periods were excluded.

Multiple studies from the same geographical area that used the same data collection were screened to avoid including duplicate data in the review. Only the publication that reported the most recent incidence trends for the same liver cancer type and population subgroup (such as sex, age group, and race/ethnicity) was included.

The titles and abstracts of all unique articles identified by the queries were independently reviewed for their eligibility by two reviewers (CH and PD). Discrepancies were discussed and resolved through consensus. The full text of all articles deemed potentially relevant and those whose abstracts and titles provided insufficient information were then retrieved for more detailed evaluation by the same two reviewers. These were categorized as “include” or “exclude” while noting the reasons for exclusion. All decisions were compared and discussed. If necessary, another author was consulted (PB).

### Critical Appraisal

A modified (14) version of the Newcastle–Ottawa Scale (NOS) (15), a risk-of-bias assessment tool for nonrandomized studies, was used for quality assessment. Studies were scored according to the extent that they met each of the nine assessed criteria using an ordinal scale to rate the risk of bias as 0 (high), 1 (intermediate), and 2 (low). Individual item scores were then summed to give a total score. Finally, the summary score for each article (average of the total scores from the two reviewers) was categorized into “high” (14–18), “moderate” (9–13.5), or “low” (<9) quality. Studies were not excluded based specifically on their quality rating.

### Data Extraction and Synthesis

For all included articles, one reviewer (CH) collected data on bibliography [author(s), year, and title], study characteristics (location, time period, design, sample size, population, and liver cancer type), relevant statistical findings for the entire cohort and/or by population subgroups, and methodological details. A second reviewer (PD) independently checked the data extract against the original source for all included articles.

Incidence was reported as age-standardized rates, with standardization being commonly performed to world or US standard populations. While trends were presented as either the annual percentage change (APC) or average APC (AAPC) over time; for simplicity, the term APC is used throughout this review for both trend measures.

Incidence trends are first presented for all liver cancers combined and then separately for each histological type. Studies are sorted by sex and age group and then by country within each of the continents (Africa, Americas, Asia, Europe, and Oceania). Some studies are repeated either in the same table or across multiple tables. Only the most recent trend is considered for studies that reported changing trends over time.

In describing trends, the terms “increase” for positive point estimates and “decrease” for negative values were used when the reported trend estimate (APC) was statistically significant (*P* < 0.05). Otherwise, if the 95% confidence interval (CI) included zero, the term “stable” was used. The term “short term” indicated that the time interval over which the trend was reported was <12 years, whereas “long term” describes trends over at least 12 years.

### Data Analysis

The published trend estimates were pooled using random-effects meta-analysis models (16) that allow for the inherent heterogeneity of observational studies. Only studies that also reported corresponding 95% CI for point estimates and sufficiently homogeneous in terms of cohort characteristics (such as age group) were retained for the meta-analysis. We also compared included vs. excluded studies by looking at the distribution of the APC point estimates and geographical location.

The pooled outcome measures were stratified by cancer type: combined liver cancers, HCC, and ICC with additional subgroup analyses by three regional categories, namely, Asia; North America/Europe/Australia comprising USA, Canada, Australia, and European countries; and Africa/South America (less developed; countries in Africa and South America), performed if there were at least four studies per stratum. Summary estimates by smaller age groups, ethnicity, or race were not generated due to wide variability in their definitions.

Heterogeneity between study-specific estimates was assessed with the *Q* and *I*^2^ statistics (17). Publication bias was evaluated by funnel plots and Egger tests (18). Sensitivity analyses were conducted by assessing the impact on the summary measure of dropping specific studies. The influence of individual studies on the pooled estimates was assessed by repeating the meta-analysis, omitting one study at a time.

Forest plots were used for graphical presentation of meta-analysis results. All analyses were performed with Stata/SE version 15 (StataCorp, TX, USA).

## Results

### Study Selection

**Figure 1:**
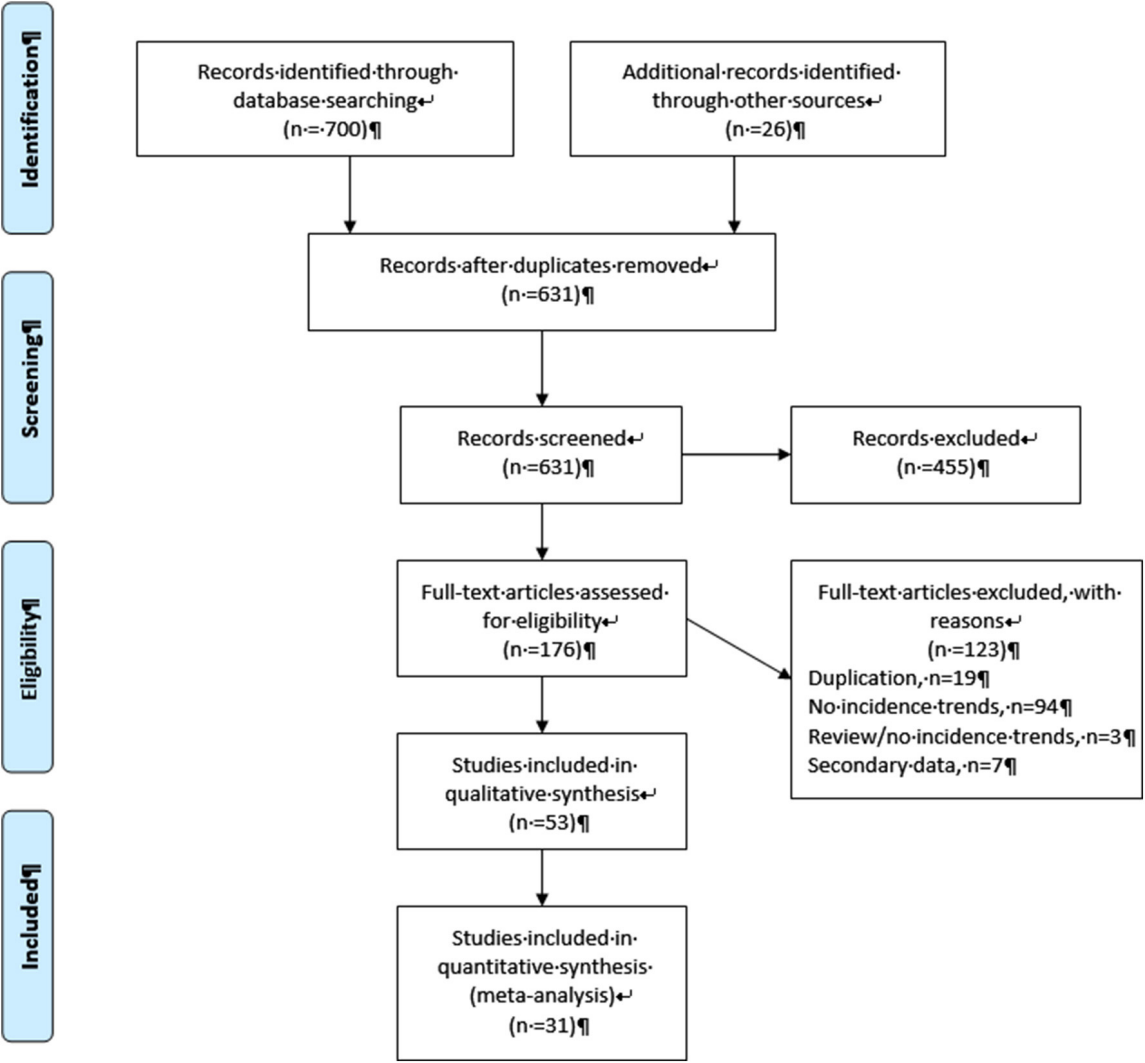
Flow diagram of study selection for the systematic review.

As shown in a PRISMA diagram (Figure 1), a total of 700 articles were identified through the search queries, with an additional 26 records found through other sources. The removal of duplicates left 631 potentially eligible records, of which 455 were excluded after initial screening of the title and abstracts as they were not relevant to the study objectives. Of the remaining 176 full-text articles that were evaluated for eligibility, 53 were retained for the review. The majority (*n* = 97) of the 123 excluded articles mentioned incidence trends in the abstract but did not specifically present quantitative estimates of these trends, while seven were based on analysis of estimated data from a secondary source and 19 articles were excluded to avoid duplication of data in the review (Supplementary File 2).

### Study Characteristics

#### Location

Sixteen of the included studies were from the USA, followed by China (9), Australia (6), South Korea (3), Japan (3), Thailand (2), and the Netherlands (2). Three more studies were also from Europe (one each from Cyprus, France, and Spain) and Asia (one each from the Philippines, Taiwan, and Lebanon), two more from the Americas (Canada and Colombia), and four from Africa (one each from Botswana, Gambia, Mozambique, and Uganda).

#### Cohort

Data for all included studies were sourced from population-based state or nationwide cancer registries, with three also accessing other administrative databases.

The median cohort size was 10,170 [interquartile range (IQR): 789–34,695]. Of the 53 studies, 28 reported trends for all liver cancers, 15 for HCC only, 7 for ICC, 1 for both HCC and ICC, and 1 for all liver cancers and HCC. One study looked at combined HCC and cholangiocarcinoma (cHCC-CC), a rare type of primary liver cancer.

The definition of “adult” varied in the included studies, with most including all ages above at least 15 years, although some restricted the upper boundary to <80 years. Eleven of 53 studies reported additional age-specific trends. Around half (*n* = 28) reported incidence trends for persons, of which 15 also presented sex-specific estimates, while the remaining studies (*n* = 25) only reported trends by sex. The only studies that reported trends by race and/or ethnicity were from the USA (10 of 16 studies).

#### Outcome Measure

All studies reported trends for age-standardized incidence rates in terms of APCs. Sixteen studies standardized age to the 2000 US population, 10 to the 1960 Segi World population, 13 to the 2000 world population, six to the 2001 Australian population, two to the European population, and 1 each to the 1985 Japanese, 2010 Korean, and 1996 Canadian populations. Three studies did not specify the standard population.

#### Quality

Twenty-two studies were rated as high quality, and the remaining 31 as moderate (Table 1, Supplementary Table 3.1). The median score was 13.5 (IQR: 13.0–14.0).

**Table 1:** Summary of included studies by study quality and overall incidence trends for the most recent time periods by sex for liver cancers combined and histological types.

			**Persons**		**Males**		**Females**	
**References^a^**	**Location**	**Quality^b^**	**Time period**	**Trend^c^-^f^**	**Time period**	**Trend^c^-^f^**	**Time period**	**Trend^c^-^f^**
**LIVER CANCERS COMBINED**^g^								
**Africa**								
Dryden-Peterson et al. (19)	BOS	Moderate	2003–2008	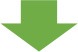	NR	NR	NR	NR
Sighoko et al. (20)	GAM	Moderate	NR	NR	1988–2006	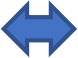	1988–2006	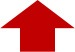
Lorenzoni et al. (21)	Maputo, MOZ	Moderate	NR	NR	1991–2008	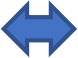	1991–2008	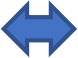
Mutyaba et al. (22)	Kampala, UGA	Moderate	1999–2008	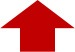	NR	NR	NR	NR
**Americas (North America)**								
Melkonian et al. (23)	USA^*h*^, ^i^	High	1999–2009	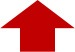	1999–2009	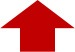	1999–2009	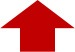
Polednak (24)	USA^h^, ^j^	Moderate	1999–2009	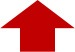	NR	NR	NR	NR
Siegel et al. (25)	USA^k^, ^l^	High	NR	NR	2003–2012	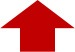	2003–2012	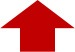
Sung et al. (26)	USA^m^, ^n^	High	1995–2014	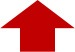	NR	NR	NR	NR
Torre et al. (27)	USA^o^, ^p^	Moderate	NR	NR	2003–2012	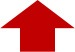	2003–2012	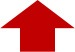
Ward et al. (28)	USA^k^	High	NR	NR	2011–2015	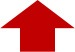	2011–2015	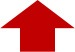
**Americas (South America)**								
Bravo et al. (29)	COL	Moderate	NR	NR	2008–2012	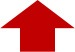	2008–2012	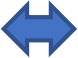
**Asia**								
Chen et al. (30)	CHN	High	NR	NR	2000–2011	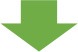	2008–2011	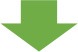
Li et al. (31)	GZ, CHN	Moderate	2004–2015	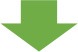	2004–2015	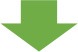	2004–2015	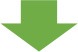
Li et al. (32)	SH, CHN	High	2002–2015	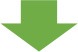	NR	NR	NR	NR
Liu et al. (33)	SH, CHN	Moderate	NR	NR	1988–2013	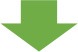	1988–2013	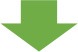
Song et al. (34)	TJ, CHN	Moderate	NR	NR	1981–2000	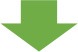	1981–2000	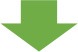
Wang et al. (35)	BJ, CHN	Moderate	NR	NR	1998–2007	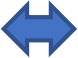	1998–2007	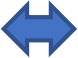
Xu et al. (36)	SZ, CHN	High	NR	NR	2001–2015	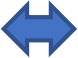	2001–2015	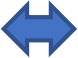
Zheng et al. (37)	CHN	Moderate	2000–2014	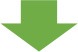				
Ito et al. (38)	Osaka, JPN	Moderate	1998–2007	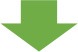	1998–2007	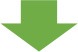	1998–2007	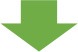
Katanoda et al. (39)	Yamagata, Fukui, and Nagasaki, JPN	Moderate	NR	NR	1992–2010	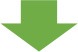	1995–2010	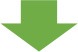
Jung et al. (40)	KOR	Moderate	1999–2012		1999–2012		1999–2012	
Shamseddine et al. (41)	LEB	Moderate	NR	NR	2003–2008	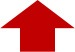	2003–2008	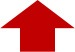
Medina et al. (42)	PHL	Moderate	NR	NR	1983–2002	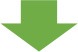	1983–2002	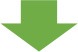
**Europe**								
Cooter et al. (43)	CYP	Moderate	NR	NR	1998–2008	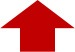	1998–2008	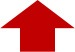
Lepage et al. (44)	FRA	Moderate	NR	NR	1980–2000	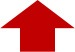	1980–2000	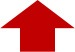
Witjes et al. (45)	NLD	Moderate	NR	NR	1989–2009	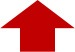	1989–2009	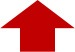
Clèries et al. (46)	ESP	Moderate	NR	NR	2000–2007	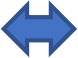	2000–2007	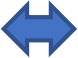
**Oceania**								
Cocker et al. (47)	AUS	Moderate	2006–2014	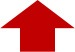	1982–2014	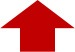	2005–2014	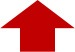
**HEPATOCELLULAR CARCINOMA**^**7**^								
**North America**								
Njei et al. (48)	USA^q^	High	2007–2011	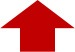	2006–2011	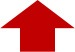	2009–2011	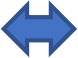
Pham et al. (49)	CA, USA	Moderate	NR	NR	1988–2012	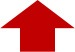	1988–2012	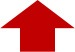
Ramirez et al. (50)	TX, USA	High	1995–2010	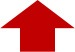	NR	NR	NR	NR
Rich et al. (51)	USA^r^	High	2010–2015^s^	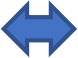	NR	NR	NR	NR
Shiels et al. (52)	USA^t^, ^u^	High	2001–2013	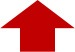	2001–2013	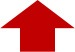	2001–2013	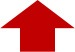
White et al. 2017 (53)	USA^h^	High	2000–2012	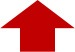	2000–2012	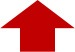	2000–2012	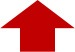
Pocobelli et al. 2008 (54)	CAN	Moderate	NR	NR	1976–2000	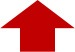	1976–2000	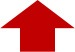
**Asia**								
Gao et al. (55)	SH, CHN	Moderate	NR	NR	1975–2005	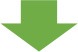	1975–2005	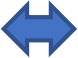
Tanaka et al. (56)	Osaka, JPN	Moderate	NR	NR	1996–2003^w^	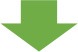	1991–2003	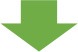
Hung et al. (57)	TWN	High	2003–2011	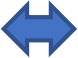	2003–2011	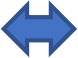	2003–2011	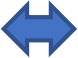
Yeesoonsang et al. (58)	Khon Kaen, THA	High	NR	NR	2007–2013	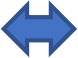	1989–2013	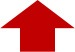
**Europe**								
Witjes et al. (45)	NLD	Moderate	1989–2009	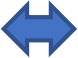	1989–2009	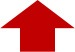	1989–2009	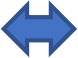
**Oceania**								
Carville et al. (59)	VIC, AUS	High	NR	NR	2004–2013	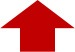	2004–2013	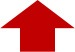
Chinnaratha et al. (60)	SA, AUS^w^	High	1996–2010	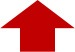	NR	NR	NR	NR
Clark et al. (61)	QLD, AUS	High	NR	NR	1996–2011	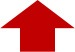	1996–2011	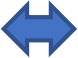
Thein et al. (62)	NSW, AUS	Moderate	1992–2007^x^	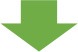	NR	NR	NR	NR
Wallace et al. (63)	AUS	High	1982–2014	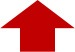	1982–2014	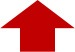	1982–2014	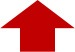
**INTRAHEPATIC CHOLANGIOCARCINOMA^g^, ^y^**								
**Americas (North America)**								
Patel and Benipal (64)	USA^h^	Moderate	2010–2015	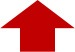	NR	NR	NR	NR
Saha et al. (65)	USA^q^	High	2003–2012	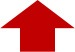	NR	NR	NR	NR
Van Dyke et al. (66)	USA^z^	High	1999–2013	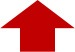	1999–2013	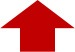	1999–2013	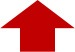
**Asia**								
Kim et al. (67)	KOR	Moderate	2006–2015	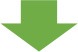				
Shin et al. (68)	KOR	Moderate	NR	NR	1999–2005	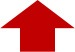	1999–2005	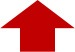
Kamsa-ard et al. (69)	Khon Kaen, THA	Moderate	2003–2009	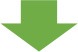	2002–2009	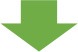	2003–2009	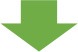
Yeesoonsang et al. (58)	Songkhla, THA	High	NR	NR	1989–2013	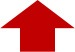	1989–2013	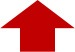
**Europe**								
Witjes et al. (70)	NLD	High	1999–2009	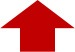				
**COMBINED HEPATOCELLULAR AND CHOLANGIOCARCINOMA**								
Wang et al. (71)	USA	High	2000–2014	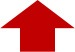	2000–2014	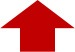	NR	NR

*MOZ, Mozambique; NLD, Netherlands; NR, Not reported, NSW, New South Wales; PHL, Philippines; QLD, Queensland; SA, South Australia; SH, Shanghai; SZ, Shenzhen; THA, Thailand; TJ, Tianjin; TWN, Taiwan; TX, Texas; UGA, Uganda; USA, United States; VIC, Victoria*.

a *All studies included adults aged at least 20 years and over unless otherwise stated*.

b *Quality categories: high (score 14–18), moderate (score 9–13.5), or low (score <9); please refer to text for further details*.

c *Trends based on annual percentage change (APC) in age-standardized incidence rate. The APC is the annual increase or decrease in incidence trends over the specified time period*.

d *Negative APC values indicate a decreasing trend, whereas positive APC values indicate an increasing trend. Stable means that the 95% confidence interval does not include zero*.

e *Increasing trends indicated by a red arrow; decreasing by a green arrow; and stable trends by a blue arrow*.

f *Only incidence trends for the most recent time period shown*.

g *Adult liver cancers were defined according to the International Classification of Disease for Oncology, Third Edition (ICD-O-3), or International Classification of Disease, Tenth Edition (ICD-10), site code (C22) and histologically as hepatocellular carcinoma (HCC) (ICD-O-3 site code C22.0, morphology codes M8170–M8175), intrahepatic cholangiocarcinoma (ICC, ICD-O-3 site code C22.1, M8160), or combined hepatocellular cholangiocarcinoma (cHCC-CC, ICD-O-3 morphology code M8180)*.

h *Based on population-based cancer incidence data from the United States Cancer Statistics registry for all 50 states in the USA and the District of Colombia*.

i *Incidence trends reported for non-Hispanic Whites and non-Hispanic American Indian/Alaska Native. Trends increased for both groups*.

j *Trends increased for persons aged 55–64 years, decreased for those aged 35–44 years, and was stable for those aged 15–34 years*.

k *Based on population-based cancer incidence data from the North American Association of Central Cancer Registries database for 47 states and the District of Puerto Rico in the USA*.

l *Incidence trends reported for non-Hispanic Whites and Hispanics. Trends increased for both groups*.

m *Based on population-based cancer incidence data from the North American Association of Central Cancer Registries database for 25 states in the USA*.

n *Trends reported by 5-year age groups*.

o *Based on population-based cancer incidence data from the North American Association of Central Cancer Registries database for 24 states and one metropolitan area (Atlanta) in the USA*.

p *Incidence trends reported for non-Hispanic Whites and Asian/Pacific Islanders. Trends increased for non-Hispanic Whites and were decreasing/stable for Asian/Pacific Islanders*.

q *Based on population-based cancer incidence data from the Surveillance, Epidemiology and End Results (SEER 18) database covering 11 states and two metropolitan areas in the USA*.

r *Based on population-based cancer incidence data from the SEER 13 database covering six states and seven regions in the USA*.

s *Overall trend only. Trends decreased for persons aged 40–59 years and increased for those older*.

t *Based on data from the SEER/Medicare-linked database*.

u *Cohort included males and females aged 66–99 years*.

v *Males and females aged 50–69 years. Trends also decreased for males (1995–2003) and females (1997–2003) aged 60–69 and remained stable for those aged 70–79 years (2000–2003)*.

w *Cohort included hepatitis B virus-infected cases*.

x *Increasing trends for hepatitis C virus-infected cases; trend remained stable for hepatitis B virus-infected cases*.

y *Studies by Kamsa-ard et al. (69) and Yeesoonsang et al. (58) include all cholangiocarcinoma cases (CHCA, ICD-O-3 site codes C22.1 and C24.0)*.

z *Based on population-based cancer incidence data from the North American Association of Central Cancer Registries database for 38 states in the USA*.

#### Meta-Analysis

Of the 53 studies, 31 (59%) were retained for the meta-analysis. Excluded studies did not present 95% CIs for the trend estimates (*n* = 16), only reported APCs by population subgroups (*n* = 5), or only reported on a rare liver cancer type (*n* = 1).

### Liver Cancers Combined

**Figure 2:**
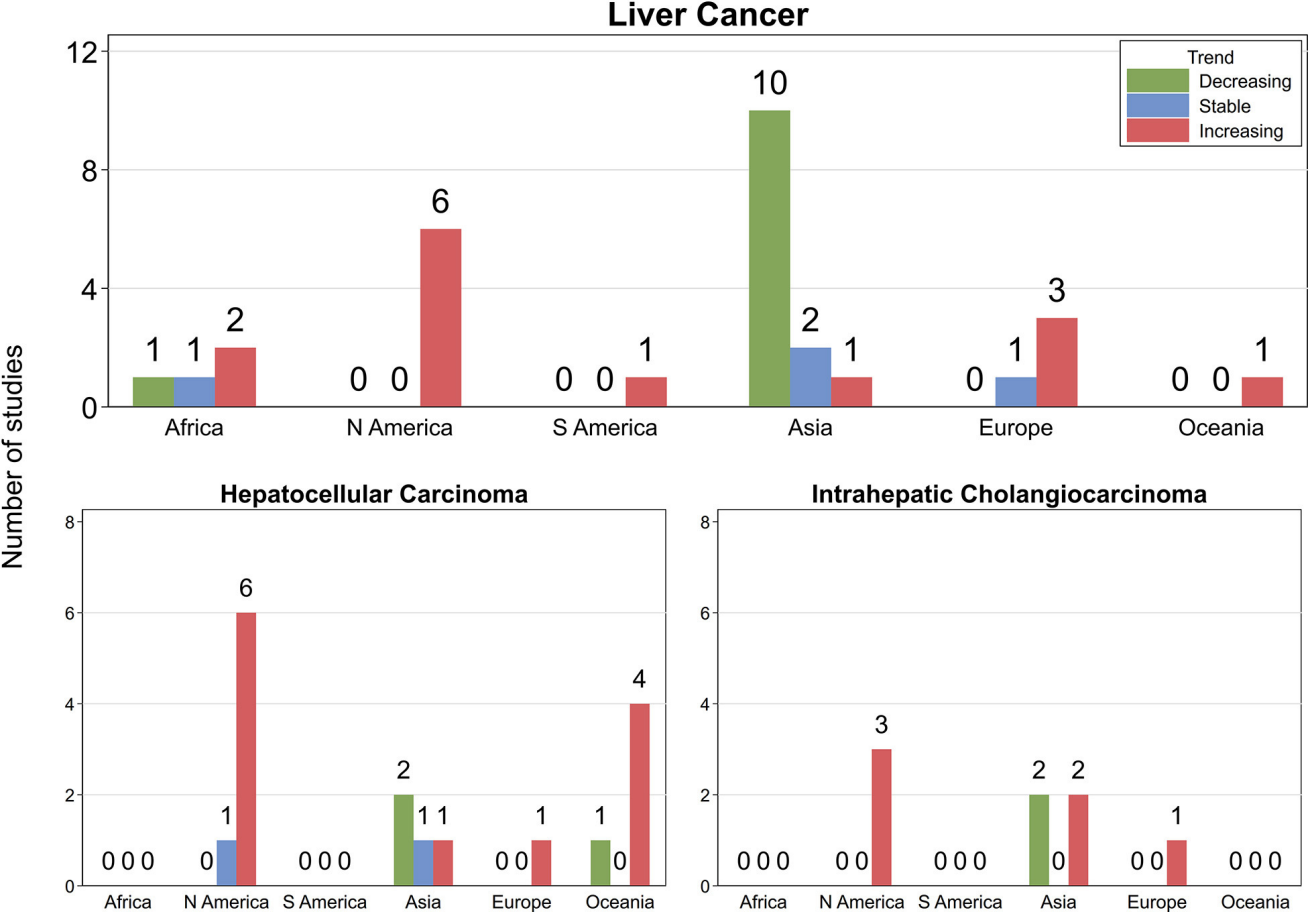
Number of studies by continent and direction of incidence trends. N America, North America; S America, South America. Green, light blue, and red bars indicate decreasing, stable, and increasing trends, respectively.

Incidence trends varied by geographical region with 10 (four high quality and six moderate) of 11 included studies from North America/Europe/Australia reporting increasing trends and one (moderate quality) stable trends. In addition, of five studies from Africa/South America, three (moderate quality) described increasing trends, one (moderate quality) stable trends, and one (moderate quality) decreasing trends. By contrast, of 13 studies from Asia, 10 (two high quality and eight moderate) described decreasing incidence trends, two (one high quality and one moderate) stable trends, and one (moderate quality) increasing trends (Figure 2, Table 1, Supplementary Table 3.2).

One study reported decreasing trends in Botswana (19) and another increasing incidence in Uganda (22) among persons until 2008. Moreover, rates remained stable over two decades to mid-2000s among males but increased among females in the Gambia (20), whereas corresponding trends were stable among both sexes in Mozambique (21).

All six studies from the USA consistently reported increasing incidence between 2011 and 2015 (28) or over recent decades (23–27). These increases were evident among persons (23, 24, 26) and by sex (23, 25, 27, 28). In Colombia, trends increased in males but were stable among females from 2008 to 2012 (29). Among both males and females in Cyprus (43), France (44), and the Netherlands (45), incidence increased over one to two decades until the 2000s but was stable in Spain (46). One study in Australia reported increasing long-term trends among persons (and by sex) until 2014 (47).

In contrast, six Chinese studies reported decreasing liver cancer incidence rates among males, females, or persons over the past one to three decades (30–34, 37) with two more (35, 36) describing stable trends from around 2000. Two studies from Japan (38, 39) and one from South Korea (40) also reported decreasing incidence across both sexes over recent decades, consistent with the decrease in the Philippines between 1983 and 2002 (42). However, sex-specific trends increased in Lebanon between 2003 and 2008 (41).

#### Trends by Age

Two studies from the USA reported that long-term trends increased over recent decades among adults ≥45 years (24, 26), decreased among those aged 35–44 (24) or 40–44 (26), and remained stable among those younger (Table 2). In contrast, incidence rates decreased across all age groups among persons aged 75 or younger over the past 10 years until around 2014 in China (31, 37).

**Table 2:** Summary of the most recent liver cancer incidence trends for included studies that present additional trend estimates by age or age and sex.

**References**	**Location**	**Age group (years)^*a*^**	**Time period**	**Trend^*b,c,d,e*^**		
				**Persons**	**Males**	**Females**
**LIVER CANCERS COMBINED**^6^						
**Americas (North America)**						
Polednak (24)	USA^*g*^	15–34	1999–2009	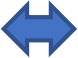	NR	NR
Polednak (24)	USA^*g*^	35–44	1999–2009	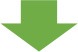	NR	NR
Polednak (24)	USA^*g*^	45+	1999–2009	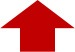	NR	NR
Sung et al. (26)	USA^*h*^	25–39	1995–2014	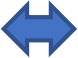	NR	NR
Sung et al. (26)	USA^*h*^	40–44	1995–2014	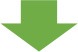	NR	NR
Sung et al. (26)	USA^*h*^	45–84	1995–2014	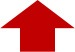	NR	NR
**Asia**						
Zheng et al. (37)	CHN	0–79	2000–2014	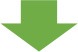	NR	NR
Zheng et al. (37)	CHN	80+	2000–2014	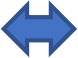	NR	NR
Li et al. (31)	SH, CHN	0–74	2004–2015	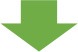	NR	NR
Li et al. (31)	SH, CHN	75+	2004–2015	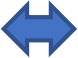	NR	NR
**HEPATOCELLULAR CARCINOMA**^6^						
**Americas (North America)**						
Pham et al. (49)	CA, USA	40–69	1988–2012	NR	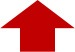	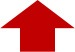
Pham et al. (49)	CA, USA	70+	1988–2012	NR	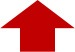	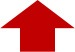
Ramirez et al. (50)	TX, USA	50–59	1995–2010	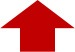	NR	NR
Ramirez et al. (50)	TX, USA	60–69	1995–2010	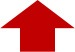	NR	NR
Ramirez et al. (50)	TX, USA	70+	1995–2010	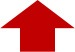	NR	NR
Rich et al. (51)	USA^*i*^	40–59^*a*^	2009–2015	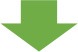	NR	NR
Rich et al. (51)	USA^*i*^	60–69	2004–2015	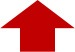	NR	NR
Rich et al. (51)	USA^*i*^	70+	1992–2015	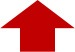	NR	NR
Shiels et al. (52)	USA^*j*^	66–75	2001–2013	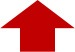	NR	NR
Shiels et al. (52)	USA^*j*^	76–85	2001–2013	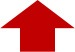	NR	NR
Shiels et al. (52)	USA^*j*^	86+	2001–2013	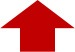	NR	NR
White et al. (53)	USA^*g*^	20–34	2000–2012	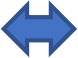	NR	NR
White et al. (53)	USA^*g*^	35–49	2000–2012	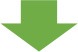	NR	NR
White et al. (53)	USA^*g*^	50+	2000–2012	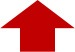	NR	NR
Pocobelli et al. (54)	CAN	20–49	1976–2000	NR	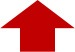	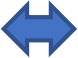
Pocobelli et al. (54)	CAN	50+	1976–2000	NR	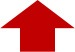	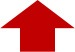
**Asia**						
Hung et al. (57)	TWN	15–64	2000–2003	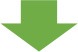	NR	NR
Hung et al. (57)	TWN	65+	2000–2003	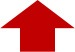	NR	NR
Tanaka et al. (56)	Osaka, JPN	50–69	1981–2003	NR	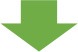	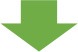
Tanaka et al. (56)	Osaka, JPN	70–79	1981–2003	NR	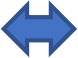	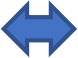
**References**	**Location**	**Age group (years)^^1^^**	**Time period**	**Trend^^2^,^3^,^4^,^5^^**		
				**Persons**	**Males**	**Females**
**Europe**						
Witjes et al. (45)	NLD	<60	1989–2009	NR	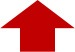	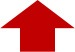
Witjes et al. (45)	NLD	60+	1989–2009	NR	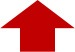	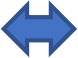
**Oceania**						
Clark et al. (61)	QLD, AUS	<50	1996–2011	NR	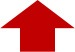	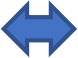
Clark et al. (61)	QLD, AUS	50–69	1996–2011	NR	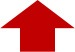	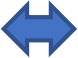
Clark et al. (61)	QLD, AUS	70+	1996–2011	NR	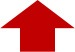	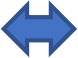
Wallace et al. (63)	AUS	45–49	1982–2014	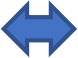	NR	NR
Wallace et al. (63)	AUS	50+	1982–2014	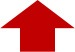	NR	NR
**INTRAHEPATIC CHOLANGIOCARCINOMA**^6^						
**Americas (North America)**						
Van Dyke et al. (66)	USA^11^	<45	1999–2013	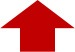	NR	NR
Van Dyke et al. (66)	USA^11^	45+	1999–2013	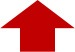	NR	NR
**Europe**						
Witjes et al. (70)	NLD	30–44	1989–2009	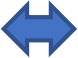	NR	NR
Witjes et al. (70)	NLD	45–59	1989–2009	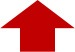	NR	NR
Witjes et al. (70)	NLD	60+	1989–2009	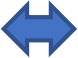	NR	NR

*AUS, Australia; CA, California; CAN, Canada; CHN, China; NLD, Netherlands; NR, Not reported; QLD, Queensland; SH, Shanghai; TWN, Taiwan; TX, Texas; USA, United States*.

1 *Only age groups for which incidence trends were reported are presented. Hence, for some studies, only certain age groups are shown*.

2 *Trends based on annual percentage change (APC) in age-standardized incidence rate. The APC is the annual increase or decrease in incidence trends over the specified time period*.

3 *Negative APC values indicate a decreasing trend, whereas positive APC values indicate an increasing trend. Stable means that the 95% confidence interval does not include zero*.

4 *Increasing trends indicated by a red arrow, decreasing trends by a green arrow, and stable trends by a blue arrow*.

5 *Only incidence trends for the most recent time period shown*.

6 *Adult liver cancers were defined according to the International Classification of Disease for Oncology, Third Edition (ICD-O-3), or International Classification of Disease, Tenth Edition (ICD-10), site code (C22) and histologically as hepatocellular carcinoma (HCC) (ICD-O-3 site code C22.0, ICD-O-3 morphology codes M8170–M8175) or intrahepatic cholangiocarcinoma (ICC, ICD-O-3 site code C22.1, M8160)*.

*^7^Based on population-based cancer incidence data from the United States Cancer Statistics registry for all 50 states in the USA and the District of Colombia*.

*^8^Based on population-based cancer incidence data from the North American Association of Central Cancer Registries database for 25 states in the USA*.

*^9^Based on population-based cancer incidence data from the Surveillance, Epidemiology and End Results (SEER 13) database covering six states and seven regions in the USA*.

*^10^Based on data from the SEER/Medicare-linked database*.

11 *Based on population-based cancer incidence data from the North American Association of Central Cancer Registries database for 38 states in the USA*.

#### Summary Estimates

**Figure 3:**
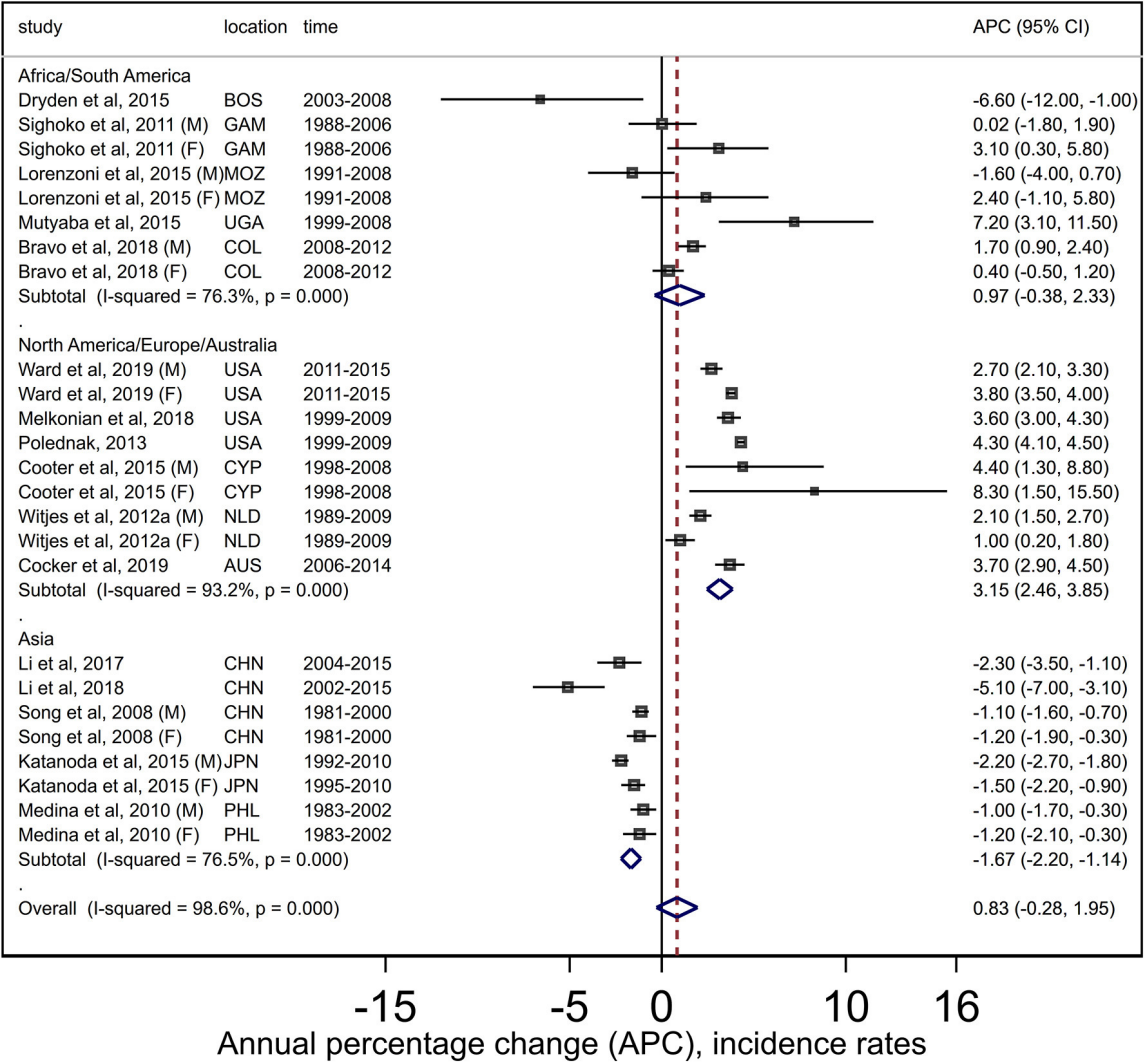
Forest plots from meta-analysis of published incidence trends for adult liver cancers. AUS, Australia; BOS, Botswana; CYP, Cyprus; GAM, Gambia; COL, Colombia; CHN, China; F, Females; JPN, Japan; M, Males; MOZ, Mozambique; NLD, Netherlands; PHL, Philippines; UGA, Uganda; USA, United States.

The pooled APC estimate for liver cancers combined was +0.8 (95% CI −0.3, +2.0) over 16 retained studies (25 APC estimates as some studies reported estimates separately for males and females). However, there was high (*I*^2^ = 98.6%) and significant (*Q* = 1,682, *P* < 0.001) heterogeneity among the studies (Figure 3), although limited publication bias was evident based on the Egger test (*P* = 0.07) and funnel plot analysis (Supplementary Figure 4.1). Summary estimates from regional subgroup analysis however indicated increasing trends (APC +3.2, 95% CI +2.5, +3.9) for North America/Europe/Australia and stable trends (APC +1.0, 95% CI −0.4, +2.3) for Africa/South America, whereas trends decreased in Asia (APC −1.7, 95% CI −2.2, −1.1). While heterogeneity remained (North America/Europe/Australia: *Q* = 117, *P* < 0.001, *I*^2^ = 76.3%; Africa/South America: *Q* = 30, *P* < 0.001, *I*^2^ = 93.2%; Asian *Q* = 30, *P* < 0.001, *I*^2^ = 76.5%), no publication bias was detected. Sensitivity analysis (results not shown) indicated that estimates were not sensitive to the inclusion/exclusion of individual studies.

### Hepatocellular Carcinoma

Of the 13 studies from non-Asian countries (all from North America/Europe/Australia), 11 (eight high quality and three moderate) reported increasing incidence rates, one (high quality) stable, and one (moderate quality) decreasing trends, whereas three (of four) studies from Asia (one high quality and two moderate) described stable and/or decreasing trends while one (high quality) noted an increase (Figure 2, Table 1, Supplementary Table 3.3).

Five studies from the USA and one from Canada all reported increasing short-term (48) or long-term trends until the early 2000s (54) or later (49, 50, 52, 53). This pattern was evident among persons (48, 50, 52, 53) and/or by sex (49, 52–54) with only one study (48) noting increasing short-term incidence for males but a stable trend among females. In addition, one study described a short-term stable trend among persons from 2010 to 2015 in the USA (51). Incidence rates increased in males but were stable among females (and persons) over two decades until 2009 in the Netherlands (45). Two Australian studies also reported increasing long-term trends among both males and females until around 2013 (59, 63) although another found that incidence increased among males but remained stable in females from 1996 to 2011 (61).

In contrast, trends decreased among males but remained stable among females from 1974 to 2005 in China (55), were stable among persons (and by sex) in Taiwan from 2003 to 2011 (57), decreased among both males and females (<69 years, 1981–2003) in Japan (56), and were stable among males (2007–2013) while increasing among females (1989–2013) in Thailand (58).

Finally, while HCC incidence rates increased in Australia among the HBV-infected population from 1996 to 2010 in one study (60), corresponding trends were stable among those with HBV while decreasing among the HCV-infected population between 1992 and 2007 (62).

#### Trends by Age

Patterns for age-specific trends were mixed (Table 2). There was a suggestion of increasing trends among older adults (≥60 years) in the USA (49–53), whereas they decreased (53) for persons aged 35–49 years and were stable for younger age groups (53) over at least 10 years from 2000 onwards. One study reported that incidence rates had decreased among both younger (40–49 years) and middle-aged (49–53, 55, 57, 59, 61, 63) adults since around mid-2000s to 2015 (51).

Age-specific rates increased by sex for those aged >50 years but only among younger males in Canada from 1976 to 2000 (54), while in the Netherlands, long-term trends (until 2009) increased among both males and females <60 years, whereas for those older, they increased among males but were stable for females (45). In contrast, rates decreased between 1981 and 2003 among both males and females <69 years of age in Japan but were stable among those aged 70–79 (56). In Taiwan, short-term trends decreased among persons <65 years but increased for those older between 2000 and 2003 (57). One Australian study described increasing long-term trends among persons aged at least 50 years (stable for ages 45–49) (63), whereas another reported increasing trends for males and stable trends for females across all age groups (61).

#### Summary Estimates

**Figure 4:**
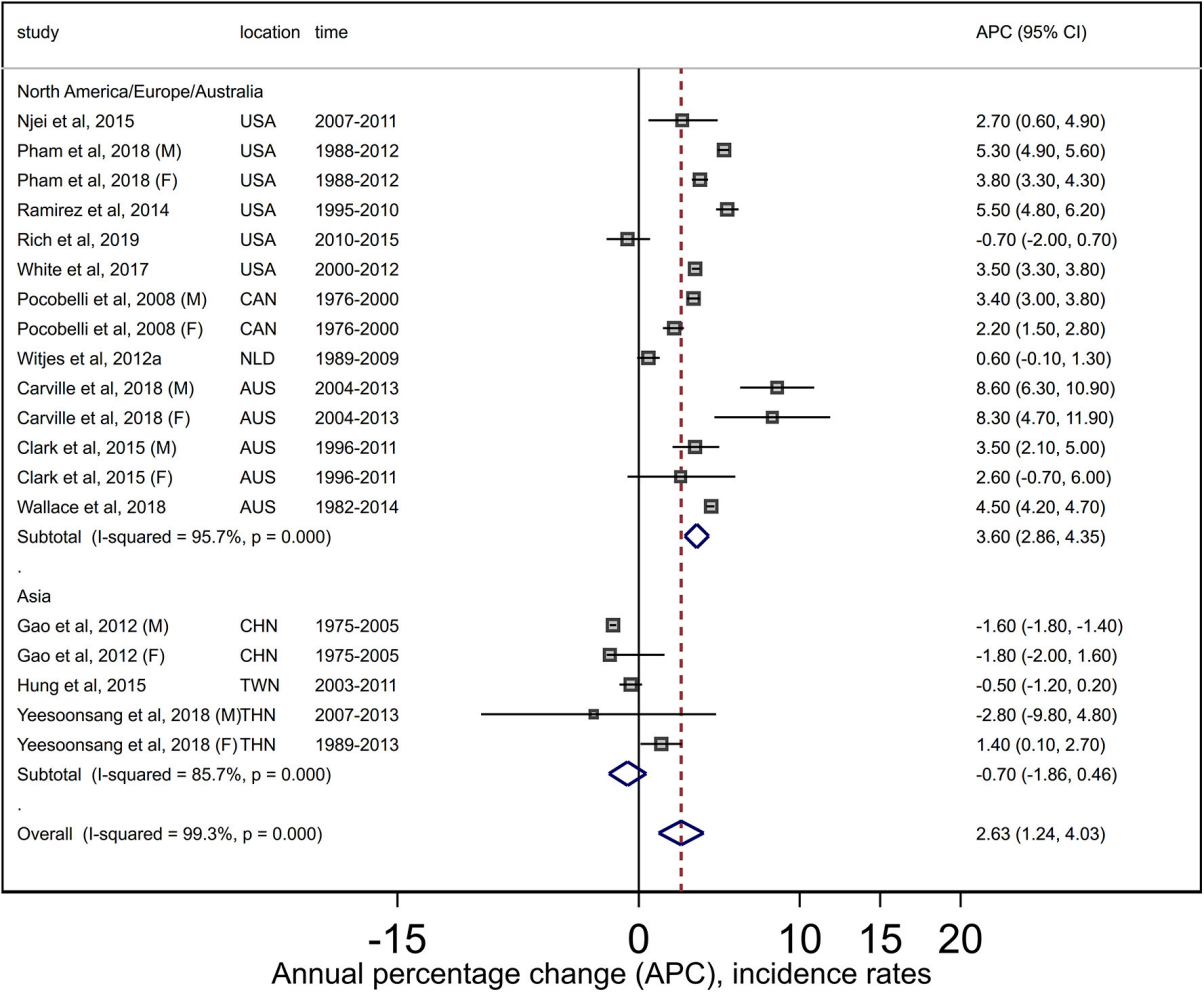
Forest plots from meta-analysis of published incidence trends for adult hepatocellular carcinoma. AUS, Australia; CAN, Canada; CHN, China; F, Females; M, Males; NLD, Netherlands; TWN, Taiwan; THA, Thailand; USA, United States.

The summary estimate of the APCs (+2.6, 95% CI +1.2, +4.0) over 13 studies (19 findings) for HCC indicated increasing incidence trends. High (*I*^2^ = 99.3%) and significant (*Q* = 2438, *P* < 0.001) heterogeneity was evident (Figure 4). No publication bias was detected (Egger *P* = 0.78, Supplementary Figure 4.1). Subgroup analysis indicated increasing trends for North America/Europe/Australia (APC +3.6, 95% CI +2.9, +4.4) and stable trends for Asia (APC −0.7, 95% CI −1.9, +0.5). While high and significant heterogeneity remained (North America/Europe/Australia: *Q* = 300, *P* < 0.001, *I*^2^ = 95.7%; Asian: *Q* = 28, *P* < 0.001, *I*^2^ = 85.7), no publication bias was detected when studies were stratified by region. Summary estimates were not sensitive to specific studies (results not shown).

### Intrahepatic Cholangiocarcinoma

Six (four high and two moderate quality) of the eight included studies reported increasing trends, and two (both moderate quality) found decreasing trends (Figure 2, Table 1, Supplementary Table 3.4). All three studies from the USA (64–66) and one from the Netherlands (70) described increasing short- or long-term trends among persons over recent decades until at least the late 2000s. While sex-specific rates increased from 1999 to 2005 (68) in South Korea, another study from the same country reported decreasing trends among persons over the decade until 2015 (67). Similarly, one study from Thailand (58) reported increasing long-term trends among both males and females over two decades until 2013, and another reported decreasing trends from 2003 to 2009 (69).

#### Trends by Age

While long-term trends increased across all ages in the USA until 2013 (66), they were stable among all age groups except those aged 45–59 years, for whom they increased from 1989 to 2009 in the Netherlands (70) (Table 2).

#### Summary Estimates

**Figure 5:**
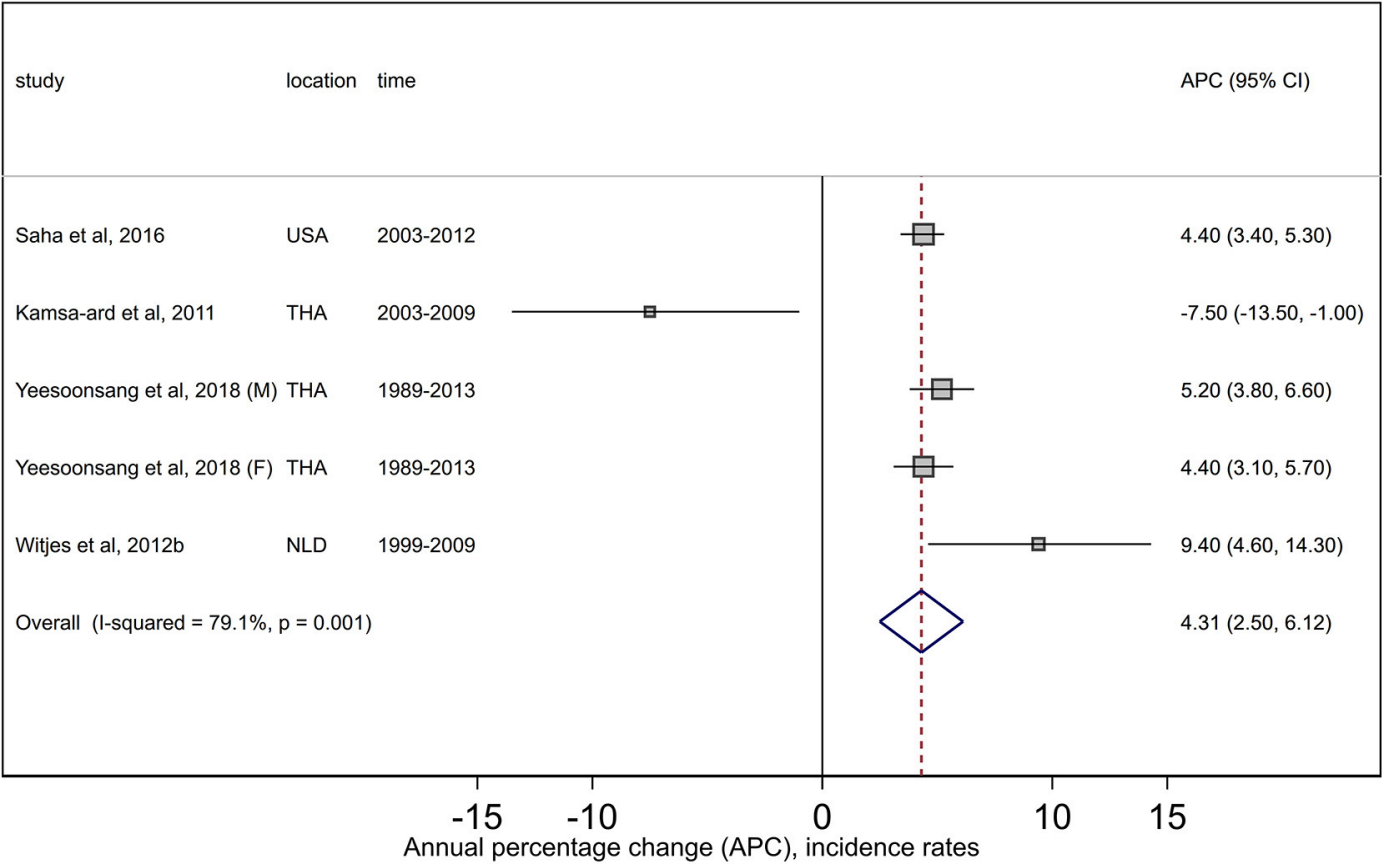
Forest plots from meta-analysis of published incidence trends for intrahepatic cholangiocarcinoma. F, Females; M, Males; NLD, Netherlands; THA, Thailand; USA, United States.

For ICC, the pooled estimate of +4.3 (95% CI +2.5, +6.1) from four retained studies indicated large increases in incidence trends. There was high (*I*^2^ = 79.1%) and significant heterogeneity (*Q* = 19.1, *P* = 0.001) between the studies (Figure 5); however, no evidence of publication bias was noted (Egger *P* = 0.68, Supplementary Figure 4.1). Trend patterns did not change during the sensitivity analysis. Summary estimates by region were not performed due to an insufficient number of studies.

### Combined Hepatocellular Cholangiocarcinoma

One study reported an increase in the incidence of cHCC-CC among both males and females over recent decades until 2014 in the USA (71).

### Trends by Race and/or Ethnicity (USA Only)

Two studies found increasing liver cancer rates overall for each analyzed racial/ethnic group (23, 25) while another reported increasing trends among non-Hispanic Whites for both sexes, whereas they decreased among Asian/Pacific Islander (API) men and were stable among API women (27) (Supplementary Table 3.5). Incidence trends over the most recent 5-year period (2011–2015) also increased among both males and females for six major racial and ethnic groups in the USA except Hispanic men and API women (both with stable trends) and API men (decreasing trends) (28).

Two studies reported increasing HCC incidence across all considered racial/ethnic groups (49, 50), and two others found increasing trends for all groups (52, 53) except APIs, where trends were either stable (52) or decreasing (53). Age-specific HCC incidence rates also increased from 1992 to 2015 among persons 60 years and older in all racial/ethnic groups except APIs aged 70–79 years, whereas they decreased among ages 40–59 years of all races/ethnicities since the mid-2000s (51). Long-term trends for ICC increased across all racial/ethnic groups until 2013 (66).

### Comparison of Excluded and Included Studies in Meta-Analysis

Visual inspection of box-and-whiskers plots for trend estimates from included and excluded studies in the meta-analysis (Supplementary Figure 4.2) showed that the median APC point estimate across studies excluded from the meta-analysis for liver cancers combined was skewed toward the right (lower or negative trends). However, the spread of the data was comparable between these two groups. For HCC and ICC, the median for the included and excluded studies was positive. Moreover, for all liver cancers (but not the two subtypes), a greater percentage of studies from the Asian region was excluded compared to non-Asian studies (62 vs. 38%, respectively—see Supplementary Figure 4.3).

## Discussion

### Summary of Main Findings

Our comprehensive systematic review found that the incidence rates for adult liver cancers combined and for HCC, the most commonly diagnosed histological subtype (4), continue to increase in Western countries. In contrast, trends decreased or were stable over recent decades for several Asian countries. These geographical patterns were reflected in summary pooled estimates from random-effects meta-analysis (16), which indicated that while trends were increasing overall and in Western countries (North America/Europe/Australia region) for both combined liver cancer and HCC, corresponding rates decreased or were stable in Asia, respectively. The different patterns may reflect geographical variation in the ratio of liver cancers attributable to HBV and HCV, with HBV being the leading etiology in Asia, whereas the opposite is true in the USA and other Western countries (8), hence the decrease of newly diagnosed HCC cases following the implementation of mass HBV immunization in countries like China and South Korea. Our results highlight the substantial variation in liver cancer incidence across countries and the need for ongoing monitoring of patterns for population subgroups within regions.

We found that incidence rates for ICC, which comprises 12–15% of all liver cancer cases (4), have increased over recent decades. Summary estimates were also suggestive of rapidly increasing trends for ICC. ICC remains, in large part, a clinical diagnosis (72), so increases in incidence rates of this cancer may reflect improved diagnostic techniques (72) and greater awareness among clinicians. This is especially so in Southeast Asian countries where ICC is endemic due to liver fluke infections (9, 10). However, recognized risk factors for ICC only account for approximately half of diagnosed cases in Western countries (10); hence, it is possible that as yet unknown etiological risk factors may also be involved.

No publication bias was detected for any of the reported meta-analysis. Sensitivity analysis suggested that the presented summary estimates are unlikely to be chance findings. Pooled estimates need to be interpreted with some caution, however, given the different periods and cohort characteristics of the included studies.

### Geographical Burden of Liver Cancer

Despite the temporal increase in liver cancer incidence in Western countries, incidence rates in Asian countries remain among the highest in the world (1). In 2018, around a quarter of all incident cases and deaths occurred in Asia, with China alone accounting for around half of the global burden (5). In addition, age-standardized rates among males and females in China were around three times higher than corresponding rates in the USA (males 27.6 vs. 10.4 per 100,000; females 9.0 vs. 3.4 per 100,000). These rates largely reflect the geographical variations in the prevalence of HBV infections, which have been estimated to be around 18% in China (73) compared to <1% in the USA (74). Hence although declining liver cancer incidence trends in Asia are a positive development, they remain a major contributor to the global liver cancer burden. In addition, the high liver cancer mortality rate (1) translates to a heavy burden on health systems that may not have resources required for intensive surgical and clinical management of HCC (7, 75).

### Proposed Drivers of Incidence Trends

Viral hepatitis infections are the key driving etiology of liver cancers, especially HCC (8). High prevalence of chronic HBV among immigrants to Western countries from endemic HBV regions such as Asia (8) was widely proposed as a contributor to increasing incidence trends (23, 27, 28, 47, 49, 52–54, 59–61, 63, 64). In the West, current increases in incidence also stem from a clinical lag time from HCV infection epidemics of the 1970s and 1980s, with these individuals now evolving to liver cirrhosis with the associated higher HCC risk (23–28, 44, 45, 47, 49, 51–54, 59, 61, 63–65). Reducing exposures to these risk factors is logically the most effective way to lower the risk of developing liver cancer, and this was reflected in the most commonly cited reasons for decreasing incidence rates being mass HBV immunization (30–34, 37, 42, 55, 57, 67), controlling HCV transmission (30, 39, 51, 56), and efficacy of antiviral therapies for hepatitis infection (19, 27, 37–39, 56, 57, 62). New anti-HCV treatments widely available in Western countries offer effective cure of HCV even for patients with advanced liver disease and reduce HCC risk by ~70% (76); however, their potential benefit has been hampered by lack of affordability and insurance coverage among certain population subgroups (73, 75, 77).

Other commonly cited nonviral contributors to increasing trends, especially in Western countries, were obesity (20, 23–25, 27, 47, 49–53, 64–66), high alcohol consumption (23, 25, 44, 49, 51–53, 59, 61, 64, 65), diabetes (20, 23–25, 27, 47, 49–53, 63–66), and NAFLD (44, 45, 47, 49, 51–53, 59, 61, 63, 66), all of which increase the risk of liver cancers (4, 6, 73, 78). Reported trends could also at least partially be an artifact of temporal changes in cancer coding and reporting practices (58, 68, 70), imaging technology (24, 43–45, 51, 58–60, 64, 65, 70), and screening (27, 45, 51, 55–57), reflecting the adoption of accepted guidelines for HCC screening and management (79, 80).

Several key risk factors for liver cancers are modifiable (7), including obesity, excessive alcohol use, smoking, and viral hepatitis infections, reflected in 71% of cases in the USA (11) being estimated to be potentially preventable. Many of the included studies highlighted the importance of continuing efforts to reduce the risks associated with viral hepatitis through primary (HBV vaccination) (23, 25, 27, 48, 49, 60) and secondary prevention efforts. The latter include screening of high-risk populations (23, 31, 47, 49, 51–53, 61) and ensuring equitable access to curative therapies for HBV/HCV infections among all population subgroups (23, 32, 37, 47, 48, 52, 53, 59). However, even if incidence of HCV-related HCC decreases in the future due to improved treatments and better control (75, 81), this may be offset by the emerging etiological importance of NAFLD as a key risk factor (77, 82). The latter reflects the continuing increase in the global prevalence of obesity (83, 84) and diabetes (84, 85), two well-established risk factors for NAFLD (86). Hence, targeted strategies for promoting healthier lifestyles (20, 23, 25, 27, 33, 36, 37, 47, 51–53, 59, 66) are also vital for reducing the global liver cancer burden. The challenge is in the development and implementation of successful strategies to achieve this goal (7, 75, 77, 87), which is especially important given the high mortality rates (1) and poor survival for liver cancer (88).

The consistently high and increasing liver cancer, and in particular HCC, incidence rates among middle-aged adults in the USA (24, 26, 49, 51–53) reflect the birth cohort effect related to HCV infection which peaked among Americans born between 1945 and 1965 through intravenous drug use during the 1960s−1980s (89). While not the primary focus of this review, racial and ethnic variations in liver cancer incidence rates in the USA were attributed at least partially to differences in the distribution of major risk factors and protective practices related to viral hepatitis control among different groups and over time (25, 27, 50, 53). For example, although APIs in the USA have had historically high liver cancer/HCC incidence rates, reflecting endemic HBV infectious among Asians, greater awareness and uptake of viral hepatitis screening and antiviral therapy may have contributed to generally decreasing trends since the mid-2000s (27, 51–53).

### Limitations

Limitations included high heterogeneity between the retained studies for the meta-analyses and the underrepresentation of studies from high-incidence countries in Africa and Asia (except Eastern and Southeastern Asia). Notably, of 31 studies included in the calculation of the pooled estimates, only three (Mozambique, Gambia, and Uganda) were from developing countries as defined by the 2019 HDI classification (12).

Although multiple databases were searched with complex queries and reference lists of identified articles were also used to maximize the completeness of those included, we acknowledge that the search terms and criteria used as well as choice of citation databases could have unintentionally resulted in some relevant articles being excluded. Only peer-reviewed published studies in English were included; hence, this review could potentially be subject to language bias.

Wide temporal and demographical variability across included studies could have impacted between-study heterogeneity. Geographical variations in the availability of high-quality cancer registry data, which could influence the reported incidence trends, mean that comparisons between (and even within) countries must be interpreted cautiously (90). Another potential limitation is the differing standard populations used for generating the ASRs.

Around two-thirds (*n* = 31, 59%) of the 53 included studies were retained in the meta-analysis; however, comparison of the point trend estimates and geographical location between the included and excluded studies suggested that the former group was broadly representative of all reviewed studies. It is nonetheless possible that the summary trend estimates may have been overestimated given that estimates from the excluded studies tended to have a right-skewed distribution in comparison to the included studies. In addition, the limited information on plausible contributors in population-based cancer registries restricted the ability of the study authors to draw firm conclusions regarding the true drivers of the reported trends.

## Conclusions

This review summarizes published estimates of global incidence trends in combined adult liver cancers by key histological types. In general, a continuing increase in incidence rates, especially among middle-aged adults, was evident across recent decades in several Western countries with historically low incidence rates. However, while trends decreased in several Asian countries, they still have the highest incidence rates worldwide, and absolute numbers remain very high. These findings highlight that liver cancer is likely to remain a major public health problem in the coming decades, albeit with a change in the distribution of cases worldwide. Demographic transition in countries where HBV is endemic offers significant hope to eventually curb the high mortality burden of HCC, though improvement is unlikely to be imminent. Emerging global trends in obesity and metabolic risk factors suggest that a new risk horizon for HCC is now evident in the West and looming in Asian and developing countries. Our findings also highlighted the impact of various public health strategies in reducing the incidence trends of viral hepatitis-related liver cancer. Design and implementation of evidence-based programs to reduce the global liver cancer burden, for example, through viral hepatitis control and lifestyle interventions, are a priority. Ongoing epidemiological surveillance is also vital to detect early shifts in incidence trends, particularly within high-risk population groups.

## Data Availability Statement

The raw data used in the meta analyses included in this article will be made available by the authors upon request.

## Author Contributions

PB, PD, JA, and DY contributed to the design of the study. PB and PD coordinated the study. PD and CH conducted the literature searches and acted as reviewers. PD drafted the manuscript. PB contributed to the initial draft of the manuscript. PB, PD, DY, JA, PC, and CH refined and approved the final version of the paper. Each author has participated sufficiently in the work and takes responsibility for appropriate portions of the content. All authors have read and have given final approval of the version to be published.

### Conflict of Interest

The authors declare that the research was conducted in the absence of any commercial or financial relationships that could be construed as a potential conflict of interest.
