# Supplementary material for: Global Trends in Incidence Rates of Primary Adult Liver Cancers: A Systematic Review and Meta-Analysis
Source: Front Oncol. 2020 Feb 28;10:171. doi: 10.3389/fonc.2020.00171 (PMC7058661; doi:10.3389/fonc.2020.00171)
Supplement: Supplementary file 1 [file Table_1.PDF]

Supplementary file 1

1 Systematic review literature search strategies

Electronic databases searched: PubMed (2008-1 September 2019), EMBASE (2008-1 September 2019) and CINAHL (2008-1 September 2019)

All search queries were conducted in a stepwise manner by breaking down each question into key concepts. Each numbered step in Tables below corresponds to the query used for an individual element such as Liver cancer or adults or outcome measures of interest. For each element, alternative terms were used to cover all possible synonyms for that component. Finally, the individual search queries were combined to create the final search query using BOOLEAN operators such as "AND" or "OR".

1.1 PUBMED

Search	Query
#1	((((((("liver neoplasms/epidemiology"[MeSH Terms]) OR adult liver cancer[MeSH Terms]) OR adult liver cancers[MeSH Terms]) OR carcinoma, hepatocellular[MeSH Terms]) OR "cholangiocarcinoma/epidemiology"[MeSH Terms]) OR "liver cancer"[Text Word] OR "liver tumour"[Text Word]
#3	((((incidence[MeSH Terms]) OR Incidence[Text Word])) AND trend[Text Word]))
#4	((((((("liver neoplasms/epidemiology"[MeSH Terms]) OR adult liver cancer[MeSH Terms]) OR adult liver cancers[MeSH Terms]) OR carcinoma, hepatocellular[MeSH Terms]) OR "cholangiocarcinoma/epidemiology"[MeSH Terms]) OR "liver cancer"[Text Word] OR "liver tumour"[Text Word] AND (((((incidence[MeSH Terms]) OR Incidence[Text Word])) AND trend [Text Word])) AND English[Language]) AND humans[MeSH Terms]) AND (("2008/01/01"[PDAT] : "2019/12/31"[PDAT])

1.2 EMBASE

Search	Query
#1	'liver cancer' OR 'liver cell carcinoma' OR 'hepatocellular carcinoma'/exp OR 'hepatocellular carcinoma'
#2	'trends'
#3	incidence OR 'incidence'/exp
#4	('incidence' OR 'incidence'/exp) AND ('liver cancer' OR 'liver cell carcinoma' OR 'hepatocellular carcinoma '/exp OR hepatocellular carcinoma) AND 'trends' AND ([article]/lim OR [article in press]/lim) AND [adult]/lim AND [english]/lim AND [2008-2019]/py

1.3 CINHAHL

Search	Query
S1	(MH "liver cancer+") OR (MH "hepatocellular carcinoma") OR TX "liver cancer" OR TX “hepatocellular carcinoma”
S2	MH adult
S3	((MH "incidence") OR TX incidence
S4	S1 AND S2 AND S3 AND LA English AND PY 2008 - 2019
