# Supplementary material for: Global Trends in Incidence Rates of Primary Adult Liver Cancers: A Systematic Review and Meta-Analysis
Source: Front Oncol. 2020 Feb 28;10:171. doi: 10.3389/fonc.2020.00171 (PMC7058661; doi:10.3389/fonc.2020.00171)
Supplement: Supplementary file 2 [file Table_2.PDF]

Supplementary file 2

1 Excluded studies with reasons for exclusion

Author, year	Reason for exclusion
Ahmed et al, 2008 (1)	Duplication, overlapping data
Altekruse et al, 2009 (2)	Duplication, overlapping data
Altekruse et al, 2014 (3)	Duplication, overlapping data
Altekruse et al, 2015 (4)	Duplication, overlapping data
Bai et al, 2018 (5)	Duplication, overlapping data
Alavi et al, 2018 (6)	No quantitative data on temporal trends
Anyiwe et al, 2016 (7)	No quantitative data on temporal trends
Are et al, 2017 (8)	Based on analysis of secondary data
Ashktorab et al, 2017 (9)	Review article, no quantitative data
Beal et al, 2018 (10)	No quantitative data on temporal trends
Beste et al, 2015 (11)	No quantitative data on temporal trends
Blakely et al, 2011 (12)	No quantitative data on temporal trends
Borie et al, 2009 (13)	No quantitative data on temporal trends
Børresen et al, 2011 (14)	No quantitative data on temporal trends
Bray et al, 2012 (15)	Based on analysis of secondary data
Card et al, 2008 (16)	No quantitative data on temporal trends
Cazzagon et al, 2013 (17)	No quantitative data on temporal trends
Center and Jemal 2011 (18)	Based on analysis of secondary data
Chaiteerakij et al, 2017(19)	No quantitative data on temporal trends
Chamberlain et al, 2013 (20)	No quantitative data on temporal trends
Chang et al, 2010 (21)	No quantitative data on temporal trends
Chokunonga et al, 2016 (22)	No quantitative data on temporal trends
Chong et al, 2013 (23)	No quantitative data on temporal trends
Cronin et al, 2018 (24)	Duplication, overlapping data
Crum-Cianflone et al, 2009 (25)	No quantitative data on temporal trends
Dal Maso et al, 2008 (26)	No quantitative data on temporal trends
Dal Maso et al, 2009 (27)	No quantitative data on temporal trends
Danos et al, 2018 (28)	No quantitative data on temporal trends
Du et al, 2014 (29)	No quantitative data on temporal trends
Edgren et al, 2008 (30)	No quantitative data on temporal trends

Author, year	Reason for exclusion
Eggert et al, 2013 (31)	Duplication, overlapping data
El-Serag 2012 (32)	Review article, no quantitative data
Engels et al, 2008 (33)	No quantitative data on temporal trends
Fidler et al, 2017 (34)	No quantitative data on temporal trends
Ford et al, 2017 (35)	No quantitative data on temporal trends
Franzetti et al, 2013 (36)	No quantitative data on temporal trends
Fwu et al, 2009 (37)	No quantitative data on temporal trends
Gao et al, 2013 (38)	Duplication, overlapping data
Gjærde et al, 2016 (39)	No quantitative data on temporal trends
Global Burden of Disease Liver Cancer et al, 2017 (40)	Based on analysis of secondary data
Gomez-Dantes et al, 2016 (41)	No quantitative data on temporal trends
Guzman et al, 2008 (42)	No quantitative data on temporal trends
Ha et al, 2016 (43)	Duplication, overlapping data
Hleyhel et al, 2014 (44)	No quantitative data on temporal trends
Ioannou et al, 2018 (45)	No quantitative data on temporal trends
Islami et al, 2017 (46)	No quantitative data on temporal trends
Jemal et al, 2013 (47)	Duplication, overlapping data
Jemal et al, 2017 (48)	Duplication, overlapping data
Jepsen et al, 2017 (49)	No quantitative data on temporal trends
Ji et al, 2012 (50)	No quantitative data on temporal trends
Joliat et al, 2015 (51)	No quantitative data on temporal trends
Jung et al, 2013 (52)	Duplication, overlapping data
Jung et al, 2014 (53)	Duplication, overlapping data
Kalyani et al, 2010 (54)	No quantitative data on temporal trends
Kim and Park 2018 (55)	No quantitative data on temporal trends
Kim et al, 2015 (56)	No quantitative data on temporal trends
Ko et al, 2018 (57)	No quantitative data on temporal trends
Kramer et al, 2017 (58)	No quantitative data on temporal trends
Lehman et al, 2008 (59)	No quantitative data on temporal trends
Long et al, 2008 (60)	No quantitative data on temporal trends
Makarova-Rusher et al, 2016 (61)	No quantitative data on temporal trends
Mayor et al, 2016 (62)	No quantitative data on temporal trends
McClune and Tong 2010 (63)	No quantitative data on temporal trends
McDonald et al, 2008 (64)	No quantitative data on temporal trends

Author, year	Reason for exclusion
Meredith et al, 2012 (65)	No quantitative data on temporal trends
Mirzaei et al, 2016 (66)	No quantitative data on temporal trends
Mitacek et al, 2008 (67)	No quantitative data on temporal trends
Mittal and El-Serag 2013 (68)	No quantitative data on temporal trends
Mittal et al, 2015 (69)	No quantitative data on temporal trends
Nordenstedt et al, 2010 (70)	Based on analysis of secondary data
Ocama et al, 2009 (71)	No quantitative data on temporal trends
Ock et al, 2018 (72)	No quantitative data on temporal trends
Oyenuga et al, 2018 (73)	No quantitative data on temporal trends
Parker et al, 2014 (74)	No quantitative data on temporal trends
Parkin et al, 2010 (75)	No quantitative data on temporal trends
Petrick et al, 2016a (76)	Based on analysis of secondary data
Petrick et al, 2016b (77)	Duplication, overlapping data
Pinero et al, 2018 (78)	No quantitative data on temporal trends
Pinter et al, 2014 (79)	No quantitative data on temporal trends
Pongnikorn et al, 2018 (80)	No quantitative data on temporal trends
Robbins et al, 2014 (81)	Duplication, overlapping data
Ryerson et al, 2016 (82)	Duplication, overlapping data
Sahasrabuddhe et al, 2012 (83)	No quantitative data on temporal trends
Said and Ghufran 2017 (84)	Review article, no quantitative data
Sanabria et al, 2013 (85)	No quantitative data on temporal trends
Sandagdorj et al, 2010 (86)	No quantitative data on temporal trends
Shiels et al, 2018 (87)	No quantitative data on temporal trends
Shiels et al, 2011 (88)	No quantitative data on temporal trends
Silverberg et al, 2015 (89)	No quantitative data on temporal trends
Simard et al, 2010 (90)	No quantitative data on temporal trends
Simard et al, 2012 (91)	Duplication, overlapping data
Singh et al, 2013 (92)	No quantitative data on temporal trends
Singh and Jemal 2017 (93)	No quantitative data on temporal trends
Sitas et al, 2013 (94)	Duplication, overlapping data
Spadea et al, 2009 (95)	No quantitative data on temporal trends
Stokkeland et al, 2010 (96)	No quantitative data on temporal trends
Su et al, 2019 (97)	No quantitative data on temporal trends
Sun et al, 2013 (98)	No quantitative data on temporal trends

Author, year	Reason for exclusion
Svircev et al, 2013 (99)	No quantitative data on temporal trends
Tangutur et al, 2011 (100)	No quantitative data on temporal trends
Taura et al, 2009 (101)	No quantitative data on temporal trends
Torre et al, 2017 (102)	No quantitative data on temporal trends
Tyson et al, 2014 (103)	No quantitative data on temporal trends
Utada et al, 2012 (104)	No quantitative data on temporal trends
Valery et al, 2018 (105)	No quantitative data on temporal trends
Von Hahn et al, 2011 (106)	No quantitative data on temporal trends
Wang et al, 2015 (107)	No quantitative data on temporal trends
Waziry et al, 2016 (108)	No quantitative data on temporal trends
Weinmann et al, 2014 (109)	No quantitative data on temporal trends
Wilson et al, 2016 (110)	No quantitative data on temporal trends
Wong et al, 2010 (111)	No quantitative data on temporal trends
Wong et al, 2015 (112)	No quantitative data on temporal trends
Wu et al, 2012 (113)	Duplication, overlapping data
Yang et al, 2018 (114)	No quantitative data on temporal trends
Yang et al, 2017 (115)	No quantitative data on temporal trends
Yang et al, 2012 (116)	No quantitative data on temporal trends
Yanik et al, 2013 (117)	No quantitative data on temporal trends
Yépez et al, 2018 (118)	No quantitative data on temporal trends
Younossi et al, 2015 (119)	No quantitative data on temporal trends
Zhang and Higashi 2009 (120)	No quantitative data on temporal trends
Zhang et al, 2011 (121)	No quantitative data on temporal trends
Zhang et al, 2015 (122)	Based on analysis of secondary data
Zimmermann et al, 2016 (123)	No quantitative data on temporal trends

2 References

1. Ahmed F, Perz JF, Kwong S, Jamison PM, Friedman C, Bell BP. National trends and disparities in the incidence of hepatocellular carcinoma, 1998-2003. *Prev Chronic Dis* (2008) 5(3):A74. PubMed PMID: 18558024; PubMed Central PMCID: PMCPMC2483571.

2. Altekruse SF, McGlynn KA, Reichman ME. Hepatocellular carcinoma incidence, mortality, and survival trends in the United States from 1975 to 2005. *J Clin Oncol* (2009) 27(9):1485-91. doi: 10.1200/JCO.2008.20.7753. PubMed PMID: 19224838; PubMed Central PMCID: PMCPMC2668555.

3. Altekruse SF, Henley SJ, Cucinelli JE, McGlynn KA. Changing hepatocellular carcinoma incidence and liver cancer mortality rates in the United States. *Am J Gastroenterol* (2014) 109(4):542-53. doi: 10.1038/ajg.2014.11.

4. Altekruse SF, Petrick JL, Rolin AI, Cuccinelli JE, Zou Z, Tatalovich Z, et al. Geographic variation of intrahepatic cholangiocarcinoma, extrahepatic cholangiocarcinoma, and hepatocellular carcinoma in the United States. *PLoS One* (2015) 10(3):e0120574. Epub 2015/04/04. doi: 10.1371/journal.pone.0120574. PubMed PMID: 25837669; PubMed Central PMCID: PMCPMC4383424.

5. Bai L, Liu Z, Fang Q, Yan Q, Shi O, Bao P, et al. The trends and projections in the incidence and mortality of liver cancer in urban Shanghai: a population-based study from 1973 to 2020. *Clin Epidemiol* (2018) 10:277-88. Epub 2018/03/23. doi: 10.2147/clep.s153951. PubMed PMID: 29563840; PubMed Central PMCID: PMCPMC5849921.

6. Alavi M, Janjua NZ, Chong M, Grebely J, Aspinall EJ, Innes H, et al. Trends in hepatocellular carcinoma incidence and survival among people with hepatitis C: An international study. *J Viral Hepat* (2018) 25(5):473-81. doi: 10.1111/jvh.12837.

7. Anyiwe K, Qiao Y, De P, Yoshida EM, Earle CC, Thein HH. Effect of socioeconomic status on hepatocellular carcinoma incidence and stage at diagnosis, a population-based cohort study. *Liver Int* (2016) 36(6):902-10. Epub 2015/10/13. doi: 10.1111/liv.12982. PubMed PMID: 26455359.

8. Are C, Meyer B, Stack A, Ahmad H, Smith L, Qian B, et al. Global trends in the burden of liver cancer. *J Surg Oncol* (2017) 115(5):591-602. Epub 2017/03/28. doi: 10.1002/jso.24518. PubMed PMID: 28345140.

9. Ashktorab H, Kupfer SS, Brim H, Carethers JM. Racial Disparity in Gastrointestinal Cancer Risk. *Gastroenterology* (2017) 153(4):910-23. Epub 2017/08/16. doi: 10.1053/j.gastro.2017.08.018. PubMed PMID: 28807841; PubMed Central PMCID: PMCPMC5623134.

10. Beal EW, Tumin D, Kabir A, Moris D, Zhang XF, Chakedis J, et al. Cohort Contributions to Race- and Gender-Specific Trends in the Incidence of Hepatocellular Carcinoma in the USA. *World J Surg* (2018) 42(3):835-40. Epub 2017/09/08. doi: 10.1007/s00268-017-4194-1. PubMed PMID: 28879603.

11. Beste LA, Leipertz SL, Green PK, Dominitz JA, Ross D, Ioannou GN. Trends in burden of cirrhosis and hepatocellular carcinoma by underlying liver disease in US veterans, 2001-2013. *Gastroenterology* (2015) 149(6):1471-82.e5; quiz e17-8. Epub 2015/08/10. doi: 10.1053/j.gastro.2015.07.056. PubMed PMID: 26255044.

12. Blakely T, Shaw C, Atkinson J, Cunningham R, Sarfati D. Social inequalities or inequities in cancer incidence? Repeated census-cancer cohort studies, New Zealand 1981-1986 to 2001-2004. *Cancer Causes Control* (2011) 22(9):1307-18. doi: 10.1007/s10552-011-9804-x.

13. Borie F, Tretarre B, Bouvier AM, Faivre J, Binder F, Launoy G, et al. Primitive liver cancers: epidemiology and geographical study in France. *Eur J Gastroenterol Hepatol* (2009) 21(9):984-9. Epub 2009/04/10. doi: 10.1097/MEG.0b013e3283293783. PubMed PMID: 19357522.

14. Børresen ML, Koch A, Biggar RJ, Andersson M, Wohlfahrt J, Ladefoged K, et al. Hepatocellular carcinoma and other liver disease among greenlanders chronically infected with hepatitis B virus: a population-based study. *JNCI: Journal of the National Cancer Institute* (2011) 103(22):1676-85. PubMed PMID: 104601693. Language: English. Entry Date: 20120323. Revision Date: 20150711. Publication Type: Journal Article.

15. Bray F, Jemal A, Grey N, Ferlay J, Forman D. Global cancer transitions according to the Human Development Index (2008-2030): a population-based study. *Lancet Oncol* (2012) 13(8):790-801. Epub 2012/06/05. doi: 10.1016/s1470-2045(12)70211-5. PubMed PMID: 22658655.

16. Card TR, Solaymani-Dodaran M, West J. Incidence and mortality of primary sclerosing cholangitis in the UK: A population-based cohort study. *J Hepatol* (2008) 48(6):939-44. doi: 10.1016/j.jhep.2008.02.017.

17. Cazzagon N, Trevisani F, Maddalo G, Giacomin A, Vanin V, Pozzan C, et al. Rise and fall of HCV-related hepatocellular carcinoma in Italy: a long-term survey from the ITA.LI.CA centres. *Liver Int* (2013) 33(9):1420-7. Epub 2013/06/14. doi: 10.1111/liv.12208. PubMed PMID: 23758775.

18. Center MM, Jemal A. International trends in liver cancer incidence rates. *Cancer Epidemiol Biomarkers Prev* (2011) 20(11):2362-8. Epub 2011/09/17. doi: 10.1158/1055-9965.epi-11-0643. PubMed PMID: 21921256.

19. Chaiteerakij R, Pan-Ngum W, Poovorawan K, Soonthornworasiri N, Treeprasertsuk S, Phaosawasdi K. Characteristics and outcomes of cholangiocarcinoma by region in Thailand: A nationwide study. *World J Gastroenterol* (2017) 23(39):7160-7. Epub 2017/11/03. doi: 10.3748/wjg.v23.i39.7160. PubMed PMID: 29093624; PubMed Central PMCID: PMCPMC5656463.

20. Chamberlain J, Sarfati D, Cunningham R, Koea J, Gurney J, Blakely T. Incidence and management of hepatocellular carcinoma among Māori and non-Māori New Zealanders. *Aust N Z J Public Health* (2013) 37(6):520-6.

21. Chang ET, Yang J, Alfaro-Velcamp T, So SKS, Glaser SL, Gomez SL. Disparities in liver cancer incidence by nativity, acculturation, and socioeconomic status in California Hispanics and Asians. *Cancer Epidemiol Biomarkers Prev* (2010) 19(12):3106-18. doi: 10.1158/1055-9965.EPI-10-0863.

22. Chokunonga E, Windridge P, Sasieni P, Borok M, Parkin DM. Black-white differences in cancer risk in Harare, Zimbabwe, during 1991-2010. *Int J Cancer* (2016) 138(6):1416-21. doi: 10.1002/ijc.29883.

23. Chong RJ, Abdullah MS, Hossain MM, Telisinghe PU, Chong VH. Rising incidence of primary liver cancer in Brunei Darussalam. *Asian Pac J Cancer Prev* (2013) 14(6):3473-7. Epub 2013/07/28. PubMed PMID: 23886131.

24. Cronin KA, Lake AJ, Scott S, Sherman RL, Noone AM, Howlader N, et al. Annual Report to the Nation on the Status of Cancer, part I: National cancer statistics. *Cancer* (2018) 124(13):2785-800. doi: 10.1002/cncr.31551. PubMed PMID: 29786848; PubMed Central PMCID: PMCPMC6033186.

25. Crum-Cianflone N, Hullsiek KH, Marconi V, Weintrob A, Ganesan A, Barthel RV, et al. Trends in the incidence of cancers among HIV-infected persons and the impact of antiretroviral therapy: A 20-year cohort study. *AIDS* (2009) 23(1):41-50. doi: 10.1097/QAD.0b013e328317cc2d.

26. Dal Maso L, Lise M, Zambon P, Crocetti E, Serraino D, Ricceri F, et al. Incidence of primary liver cancer in Italy between 1988 and 2002: An age-period-cohort analysis. *Eur J Cancer* (2008) 44(2):285-92. doi: 10.1016/j.ejca.2007.11.009.

27. Dal Maso L, Polesel J, Serraino D, Lise M, Piselli P, Falcini F, et al. Pattern of cancer risk in persons with AIDS in Italy in the HAART era. *Br J Cancer* (2009) 100(5):840-7. Epub 2009/02/19. doi: 10.1038/sj.bjc.6604923. PubMed PMID: 19223894; PubMed Central PMCID: PMCPMC2653754.

28. Danos D, Leonardi C, Gilliland A, Shankar S, Srivastava RK, Simonsen N, et al. Increased Risk of Hepatocellular Carcinoma Associated With Neighborhood Concentrated Disadvantage. *Front Oncol* (2018)

8:375. doi: 10.3389/fonc.2018.00375. PubMed PMID: 30254987; PubMed Central PMCID: PMCPMC6141716.

29. Du LB, Li HZ, Wang XH, Zhu C, Liu QM, Li QL, et al. Analysis of cancer incidence in Zhejiang cancer registry in China during 2000 to 2009. *Asian Pac J Cancer Prev* (2014) 15(14):5839-43.

30. Edgren G, Reilly M, Hjalgrim H, Tran TN, Rostgaard K, Adami J, et al. Donation frequency, iron loss, and risk of cancer among blood donors. *JNCI: Journal of the National Cancer Institute* (2008) 100(8):572-9. PubMed PMID: 105736775. Language: English. Entry Date: 20080606. Revision Date: 20150711. Publication Type: Journal Article.

31. Eggert T, McGlynn KA, Duffy A, Manns MP, Greten TF, Altekruse SF. Fibrolamellar hepatocellular carcinoma in the USA, 2000–2010: A detailed report on frequency, treatment and outcome based on the surveillance, epidemiology, and end results database. *United European Gastroenterology Journal* (2013) 1(5):351-7. doi: 10.1177/2050640613501507.

32. El-Serag HB. Epidemiology of viral hepatitis and hepatocellular carcinoma. *Gastroenterology* (2012) 142(6):1264-73.e1. Epub 2012/04/28. doi: 10.1053/j.gastro.2011.12.061. PubMed PMID: 22537432; PubMed Central PMCID: PMCPMC3338949.

33. Engels EA, Biggar RJ, Hall HI, Cross H, Crutchfield A, Finch JL, et al. Cancer risk in people infected with human immunodeficiency virus in the United States. *Int J Cancer* (2008) 123(1):187-94. doi: 10.1002/ijc.23487.

34. Fidler MM, Gupta S, Soerjomataram I, Ferlay J, Steliarova-Foucher E, Bray F. Cancer incidence and mortality among young adults aged 20-39 years worldwide in 2012: a population-based study. *Lancet Oncol* (2017) 18(12):1579-89. Epub 2017/11/08. doi: 10.1016/s1470-2045(17)30677-0. PubMed PMID: 29111259.

35. Ford MM, Ivanina E, Desai P, Highfield L, Qiao B, Schymura MJ, et al. Geographic epidemiology of hepatocellular carcinoma, viral hepatitis, and socioeconomic position in New York City. *Cancer Causes Control* (2017) 28(7):779-89. Epub 2017/06/03. doi: 10.1007/s10552-017-0897-8. PubMed PMID: 28573469.

36. Franzetti M, Adorni F, Parravicini C, Vergani B, Antinori S, Milazzo L, et al. Trends and predictors of non-AIDS-defining cancers in men and women with HIV infection: A single-institution retrospective study before and after the introduction of HAART. *J Acquir Immune Defic Syndr* (2013) 62(4):414-20. doi: 10.1097/QAI.0b013e318282a189.

37. Fwu CW, Chien YC, Kirk GD, Nelson KE, You SL, Kuo HS, et al. Hepatitis B virus infection and hepatocellular carcinoma among parous Taiwanese women: nationwide cohort study. *JNCI: Journal of the National Cancer Institute* (2009) 101(14):1019-27. doi: jnci/djp146. PubMed PMID: 105384103. Language: English. Entry Date: 20090807. Revision Date: 20150711. Publication Type: Journal Article.

38. Gao J, Xie L, Chen WQ, Zhang SW, Wu QJ, Yang Y, et al. Rural-urban, sex variations, and time trend of primary liver cancer incidence in China, 1988-2005. *Eur J Cancer Prev* (2013) 22(5):448-54. doi: 10.1097/CEJ.0b013e32835de82a.

39. Gjærde LI, Shepherd L, Jablonowska E, Lazzarin A, Rougemont M, Darling K, et al. Trends in Incidences and Risk Factors for Hepatocellular Carcinoma and Other Liver Events in HIV and Hepatitis C Virus-coinfected Individuals from 2001 to 2014: A Multicohort Study. *Clin Infect Dis* (2016) 63(6):821-9. doi: 10.1093/cid/ciw380.

40. Global Burden of Disease Liver Cancer C, Akinyemiju T, Abera S, Ahmed M, Alam N, Alemayohu MA, et al. The Burden of Primary Liver Cancer and Underlying Etiologies From 1990 to 2015 at the Global, Regional, and National Level: Results From the Global Burden of Disease Study 2015. *JAMA Oncol* (2017) 3(12):1683-91. doi: 10.1001/jamaoncol.2017.3055. PubMed PMID: 28983565; PubMed Central PMCID: PMCPMC5824275.

41. Gomez-Dantes H, Lamadrid-Figueroa H, Cahuana-Hurtado L, Silverman-Retana O, Montero P, Gonzalez-Robledo MC, et al. The burden of cancer in Mexico, 1990-2013. *Salud Publica Mex* (2016) 58(2):118-31. Epub 2016/08/25. PubMed PMID: 27557370.

42. Guzman G, Brunt EM, Petrovic LM, Chejfec G, Layden TJ, Cotler SJ. Does nonalcoholic fatty liver disease predispose patients to hepatocellular carcinoma in the absence of cirrhosis? *Arch Pathol Lab Med* (2008) 132(11):1761-6. PubMed PMID: 105569793. Language: English. Entry Date: 20090102. Revision Date: 20150711. Publication Type: Journal Article.

43. Ha J, Yan M, Aguilar M, Bhuket T, Tana MM, Liu B, et al. Race/ethnicity-specific disparities in cancer incidence, burden of disease, and overall survival among patients with hepatocellular carcinoma in the United States. *Cancer* (2016) 122(16):2512-23. doi: 10.1002/cncr.30103.

44. Hleyhel M, Hleyhel M, Bouvier AM, Belot A, Tattevin P, Pacanowski J, et al. Risk of non-AIDS-defining cancers among HIV-1-infected individuals in France between 1997 and 2009: results from a French cohort. *AIDS* (2014) 28(14):2109-18. Epub 2014/09/30. doi: 10.1097/qad.0000000000000382. PubMed PMID: 25265077.

45. Ioannou GN, Green P, Lowy E, Mun EJ, Berry K. Differences in hepatocellular carcinoma risk, predictors and trends over time according to etiology of cirrhosis. *PLoS One* (2018) 13(9). doi: 10.1371/journal.pone.0204412.

46. Islami F, Miller KD, Siegel RL, Fedewa SA, Ward EM, Jemal A. Disparities in liver cancer occurrence in the United States by race/ethnicity and state. *CA Cancer J Clin* (2017) 67(4):273-89. Epub 2017/06/07. doi: 10.3322/caac.21402. PubMed PMID: 28586094.

47. Jemal A, Simard EP, Dorell C, Noone AM, Markowitz LE, Kohler B, et al. Annual report to the nation on the status of cancer, 1975-2009, featuring the burden and trends in human papillomavirus (HPV)-associated cancers and HPV vaccination coverage levels. *J Natl Cancer Inst* (2013) 105(3):175-201. doi: 10.1093/jnci/djs491.

48. Jemal A, Ward EM, Johnson CJ, Cronin KA, Ma J, Ryerson B, et al. Annual Report to the Nation on the Status of Cancer, 1975-2014, Featuring Survival. *J Natl Cancer Inst* (2017) 109(9). Epub 2017/04/05. doi: 10.1093/jnci/djx030. PubMed PMID: 28376154; PubMed Central PMCID: PMCPMC5409140.

49. Jepsen P, Andersen MW, Villadsen GE, Ott P, Vilstrup H. Time-trends in incidence and prognosis of hepatocellular carcinoma in Denmark: A nationwide register-based cohort study. *Liver Int* (2017) 37(6):871-8. Epub 2016/12/13. doi: 10.1111/liv.13340. PubMed PMID: 27943604.

50. Ji J, Sundquist K, Sundquist J. A population-based study of hepatitis d virus as potential risk factor for hepatocellular carcinoma. *JNCI: Journal of the National Cancer Institute* (2012) 104(10):790-2. PubMed PMID: 104446997. Language: English. Entry Date: 20120720. Revision Date: 20150711. Publication Type: Journal Article.

51. Joliat GR, Hahnloser D, Demartines N, Schäfer M. Future development of gastrointestinal cancer incidence and mortality rates in Switzerland: A tumour registry- and population-based projection up to 2030. *Swiss Med Wkly* (2015) 145. doi: 10.4414/smw.2015.14188.

52. Jung KW, Won YJ, Kong HJ, Oh CM, Seo HG, Lee JS. Cancer statistics in Korea: Incidence, mortality, survival and prevalence in 2010. *Cancer Res Treat* (2013) 45(1):1-14. doi: 10.4143/crt.2013.45.1.1.

53. Jung K, Won Y, Kong H, Oh C, Lee DH, Lee JS. Cancer statistics in Korea: Incidence, mortality, survival and prevalence in 2011. *Cancer Res Treat* (2014) 46(2):109-23. doi: 10.4143/crt.2014.46.2.109.

54. Kalyani R, Das S, Kumar ML. Spectrum of gastro-intestinal cancers--a ten-year study. *J Indian Med Assoc* (2010) 108(10):659-62. Epub 2011/04/23. PubMed PMID: 21510549.

55. Kim BH, Park JW. Epidemiology of liver cancer in South Korea. *Clin Mol Hepatol* (2018) 24(1):1-9. doi: 10.3350/cmh.2017.0112. PubMed PMID: 29249129; PubMed Central PMCID: PMCPMC5875192.

56. Kim GA, Lee HC, Kim MJ, Ha Y, Park EJ, An J, et al. Incidence of hepatocellular carcinoma after HBsAg seroclearance in chronic hepatitis B patients: a need for surveillance. *J Hepatol* (2015) 62(5):1092-9. Epub 2014/12/03. doi: 10.1016/j.jhep.2014.11.031. PubMed PMID: 25445399.

57. Ko KP, Shin A, Cho S, Park SK, Yoo KY. Environmental contributions to gastrointestinal and liver cancer in the Asia-Pacific region. *J Gastroenterol Hepatol* (2018) 33(1):111-20. Epub 2017/09/30. doi: 10.1111/jgh.14005. PubMed PMID: 28960448.

58. Kramer JR, El-Serag HB, Taylor TJ, White DL, Asch SM, Frayne SM, et al. Hepatitis C virus-related complications are increasing in women veterans: A national cohort study. *J Viral Hepat* (2017) 24(11):955-65. Epub 2017/08/18. doi: 10.1111/jvh.12728. PubMed PMID: 28815822; PubMed Central PMCID: PMCPMC5638671.

59. Lehman EM, Soliman AS, Ismail K, Hablas A, Seifeldin IA, Ramadan M, et al. Patterns of hepatocellular carcinoma incidence in Egypt from a population-based cancer registry. *Hepatol Res* (2008) 38(5):465-73. doi: 10.1111/j.1872-034X.2007.00299.x.

60. Long JL, Engels EA, Moore RD, Gebo KA. Incidence and outcomes of malignancy in the HAART era in an urban cohort of HIV-infected individuals. *AIDS* (2008) 22(4):489-96. doi: 10.1097/QAD.0b013e3282f47082.

61. Makarova-Rusher OV, Altekruse SF, McNeel TS, Ulahannan S, Duffy AG, Graubard BI, et al. Population attributable fractions of risk factors for hepatocellular carcinoma in the United States. *Cancer* (2016) 122(11):1757-65. Epub 2016/03/22. doi: 10.1002/cncr.29971. PubMed PMID: 26998818; PubMed Central PMCID: PMCPMC5548177.

62. Mayor AM, Santiago-Rodriguez EJ, Rios-Olivares E, Tortolero-Luna G, Hunter-Mellado RF. Malignancies trends in a hispanic cohort of HIV persons in puerto rico before and after cART. *International Journal of Cancer Research* (2016) 12(2):92-100. doi: 10.3923/ijcr.2016.92.100.

63. McClune AC, Tong MJ. Chronic hepatitis B and hepatocellular carcinoma. *Clin Liver Dis* (2010) 14(3):461-76. Epub 2010/07/20. doi: 10.1016/j.cld.2010.05.009. PubMed PMID: 20638025.

64. McDonald SA, Hutchinson SJ, Bird SM, Robertson C, Mills PR, Dillon JF, et al. A record-linkage study of the development of hepatocellular carcinoma in persons with hepatitis C infection in Scotland. *Br J Cancer* (2008) 99(5):805-10. doi: 10.1038/sj.bjc.6604563.

65. Meredith I, Sarfati D, Ikeda T, Blakely T. Cancer in Pacific people in New Zealand. *Cancer Causes Control* (2012) 23(7):1173-84. Epub 2012/05/24. doi: 10.1007/s10552-012-9986-x. PubMed PMID: 22618362.

66. Mirzaei M, Ghoncheh M, Pournamdar Z, Soheilipour F, Salehiniya H. Incidence and Trend of Liver Cancer in Iran. *J Coll Physicians Surg Pak* (2016) 26(4):306-9. Epub 2016/04/22. doi: 2297. PubMed PMID: 27097703.

67. Mitacek EJ, Brunnemann KD, Suttajit M, Caplan LS, Gagna CE, Bhothisuwan K, et al. Geographic distribution of liver and stomach cancers in thailand in relation to estimated dietary intake of nitrate, nitrite, and nitrosodimethylamine. *Nutr Cancer* (2008) 60(2):196-203. doi: 10.1080/01635580701649636.

68. Mittal S, El-Serag HB. Epidemiology of hepatocellular carcinoma: consider the population. *J Clin Gastroenterol* (2013) 47 Suppl:S2-6. Epub 2013/05/02. doi: 10.1097/MCG.0b013e3182872f29. PubMed PMID: 23632345; PubMed Central PMCID: PMCPMC3683119.

69. Mittal S, Sada YH, El-Serag HB, Kanwal F, Duan Z, Temple S, et al. Temporal trends of nonalcoholic fatty liver disease-related hepatocellular carcinoma in the veteran affairs population. *Clin Gastroenterol Hepatol* (2015) 13(3):594-601 e1. doi: 10.1016/j.cgh.2014.08.013. PubMed PMID: 25148760; PubMed Central PMCID: PMCPMC4333060.

70. Nordenstedt H, White DL, El-Serag HB. The changing pattern of epidemiology in hepatocellular carcinoma. *Dig Liver Dis* (2010) 42 Suppl 3:S206-14. Epub 2010/06/25. doi: 10.1016/s1590-8658(10)60507-5. PubMed PMID: 20547305; PubMed Central PMCID: PMCPMC3392755.

71. Ocama P, Nambooze S, Opio CK, Shiels MS, Wabinga HR, Kirk GD. Trends in the incidence of primary liver cancer in Central Uganda, 1960-1980 and 1991-2005. *Br J Cancer* (2009) 100(5):799-802. doi: 10.1038/sj.bjc.6604893.

72. Ock M, Choi WJ, Jo MW. Trend analysis of major cancer statistics according to sex and severity levels in Korea. *PLoS One* (2018) 13(9). doi: 10.1371/journal.pone.0203110.

73. Oyenuga M, Yang JK, Prizment AE, Bushhouse S, Demerath EW, Spector LG. Cancer patterns in Hmong in Minnesota, 2000 to 2012. *Cancer* (2018) 124(17):3560-6. doi: 10.1002/cncr.31592. PubMed PMID: 29975407.

74. Parker C, Tong SY, Dempsey K, Condon J, Sharma SK, Chen JW, et al. Hepatocellular carcinoma in Australia's Northern Territory: high incidence and poor outcome. *Med J Aust* (2014) 201(8):470-4. Epub 2014/10/22. PubMed PMID: 25332035.

75. Parkin DM, Nambooze S, Wabwire-Mangen F, Wabinga HR. Changing cancer incidence in Kampala, Uganda, 1991-2006. *Int J Cancer* (2010) 126(5):1187-95. doi: 10.1002/ijc.24838.

76. Petrick JL, Braunlin M, Laversanne M, Valery PC, Bray F, McGlynn KA. International trends in liver cancer incidence, overall and by histologic subtype, 1978–2007. *Int J Cancer* (2016) 139(7):1534-45. doi: 10.1002/ijc.30211.

77. Petrick JL, Kelly SP, Altekruse SF, McGlynn KA, Rosenberg PS. Future of Hepatocellular Carcinoma Incidence in the United States Forecast Through 2030. *J Clin Oncol* (2016) 34(15):1787-94. Epub 2016/04/06. doi: 10.1200/jco.2015.64.7412. PubMed PMID: 27044939; PubMed Central PMCID: PMCPMC4966339.

78. Pinero F, Costa P, Boteon YL, Duque SH, Marciano S, Anders M, et al. A changing etiologic scenario in liver transplantation for hepatocellular carcinoma in a multicenter cohort study from Latin America. *Clin Res Hepatol Gastroenterol* (2018) 42(5):443-52. doi: 10.1016/j.clinre.2018.03.014. PubMed PMID: 29773419.

79. Pinter M, Hucke F, Zielonke N, Waldhör T, Trauner M, Peck-Radosavljevic M, et al. Incidence and mortality trends for biliary tract cancers in Austria. *Liver International* (2014) 34(7):1102-8. doi: 10.1111/liv.12325.

80. Pongnikorn D, Daoprasert K, Waisri N, Laversanne M, Bray F. Cancer incidence in northern Thailand: Results from six population-based cancer registries 1993–2012. *Int J Cancer* (2018) 142(9):1767-75. doi: 10.1002/ijc.31203.

81. Robbins HA, Shiels MS, Pfeiffer RM, Engels EA. Epidemiologic contributions to recent cancer trends among HIV-infected people in the United States. *AIDS* (2014) 28(6):881-90. doi: 10.1097/QAD.0000000000000163.

82. Ryerson AB, Eheman CR, Altekruse SF, Ward JW, Jemal A, Sherman RL, et al. Annual Report to the Nation on the Status of Cancer, 1975-2012, featuring the increasing incidence of liver cancer. *Cancer* (2016) 122(9):1312-37. doi: 10.1002/cncr.29936. PubMed PMID: 26959385; PubMed Central PMCID: PMCPMC4840031.

83. Sahasrabuddhe VV, Shiels MS, McGlynn KA, Engels EA. The risk of hepatocellular carcinoma among individuals with acquired immunodeficiency syndrome in the United States. *Cancer* (2012) 118(24):6226-33. Epub 2012/06/28. doi: 10.1002/cncr.27694. PubMed PMID: 22736272; PubMed Central PMCID: PMCPMC3965349.

84. Said A, Ghufran A. Epidemic of non-alcoholic fatty liver disease and hepatocellular carcinoma. *World J Clin Oncol* (2017) 8(6):429-36. Epub 2018/01/02. doi: 10.5306/wjco.v8.i6.429. PubMed PMID: 29291167; PubMed Central PMCID: PMCPMC5740098.

85. Sanabria AJ, Dion R, Lúcar E, Soto JC. Evolution of the determinants of chronic liver disease in Quebec. *Chronic Dis Inj Can* (2013) 33(3):137-45.

86. Sandagdorj T, Sanjaajamts E, Tudev U, Oyunchimeg D, Ochir C, Roder D. Cancer incidence and mortality in Mongolia - National Registry Data. *Asian Pac J Cancer Prev* (2010) 11(6):1509-14. Epub 2011/02/23. PubMed PMID: 21338189.

87. Shiels MS, Islam JY, Rosenberg PS, Hall HI, Jacobson E, Engels EA. Projected Cancer Incidence Rates and Burden of Incident Cancer Cases in HIV-Infected Adults in the United States Through 2030. *Ann Intern Med* (2018) 168(12):866-73. doi: 10.7326/M17-2499.

88. Shiels MS, Pfeiffer RM, Gail MH, Hall HI, Li J, Chaturvedi AK, et al. Cancer burden in the HIV-infected population in the United States. *J Natl Cancer Inst* (2011) 103(9):753-62. doi: 10.1093/jnci/djr076.

89. Silverberg MJ, Lau B, Achenbach CJ, Jing Y, Althoff KN, D'Souza G, et al. Cumulative Incidence of Cancer Among Persons With HIV in North America: A Cohort Study. *Ann Intern Med* (2015) 163(7):507-18. doi: 10.7326/M14-2768.

90. Simard EP, Pfeiffer RM, Engels EA. Spectrum of cancer risk late after AIDS onset in the United States. *Arch Intern Med* (2010) 170(15):1337-45. doi: 10.1001/archinternmed.2010.253. PubMed PMID: 105075086. Language: English. Entry Date: 20100917. Revision Date: 20150711. Publication Type: Journal Article.

91. Simard EP, Ward EM, Siegel R, Jemal A. Cancers with increasing incidence trends in the United States: 1999 through 2008. *CA Cancer J Clin* (2012) 62(2):118-28. Epub 2012/01/28. doi: 10.3322/caac.20141. PubMed PMID: 22281605.

92. Singh G, Siahpush M, Altekruse S. Time Trends in Liver Cancer Mortality, Incidence, and Risk Factors by Unemployment Level and Race/Ethnicity, United States, 1969-2011. *J Community Health* (2013) 38(5):926-40. doi: 10.1007/s10900-013-9703-z. PubMed PMID: 104223975. Language: English. Entry Date: 20130911. Revision Date: 20171010. Publication Type: Journal Article.

93. Singh GK, Jemal A. Socioeconomic and Racial/Ethnic Disparities in Cancer Mortality, Incidence, and Survival in the United States, 1950-2014: Over Six Decades of Changing Patterns and Widening Inequalities. *J Environ Public Health* (2017) 2017, Article ID 281937, 10.1155/2017/2819372(doi 10.1155/2017/2819372). doi: 10.1155/2017/2819372.

94. Sitas F, Gibberd A, Kahn C, Weber MF, Chiew M, Supramaniam R, et al. Cancer incidence and mortality in people aged less than 75 years: changes in Australia over the period 1987-2007. *Cancer Epidemiol* (2013) 37(6):780-7. Epub 2013/11/05. doi: 10.1016/j.canep.2013.09.010. PubMed PMID: 24183782.

95. Spadea T, D'Errico A, Demaria M, Faggiano F, Pasian S, Zanetti R, et al. Educational inequalities in cancer incidence in Turin, Italy. *Eur J Cancer Prev* (2009) 18(3):169-78. doi: 10.1097/CEJ.0b013e3283265bc9.

96. Stokkeland K, Ebrahim F, Ekbom A. Increased Risk of Esophageal Varices, Liver Cancer, and Death in Patients With Alcoholic Liver Disease. *Alcoholism: Clinical & Experimental Research* (2010) 34(11):1993-9. doi: 10.1111/j.1530-0277.2010.01289.x. PubMed PMID: 105009790. Language: English. Entry Date: 20101116. Revision Date: 20150711. Publication Type: Journal Article.

97. Su SY, Chiang CJ, Yang YW, Lee WC. Secular trends in liver cancer incidence from 1997 to 2014 in Taiwan and projection to 2035: An age-period-cohort analysis. *J Formos Med Assoc* (2019) 118(1 Pt 3):444-9. doi: 10.1016/j.jfma.2018.07.001. PubMed PMID: 30017535.

98. Sun Z, Chen T, Thorgeirsson SS, Zhan Q, Chen J, Park JH, et al. Dramatic reduction of liver cancer incidence in young adults: 28 Year follow-up of etiological interventions in an endemic area of China. *Carcinogenesis* (2013) 34(8):1800-5. doi: 10.1093/carcin/bgt007.

99. Svircev Z, Drobac D, Tokodi N, Vidovic M, Simeunovic J, Miladinov-Mikov M, et al. Epidemiology of primary liver cancer in Serbia and possible connection with cyanobacterial blooms. *J Environ Sci Health C Environ Carcinog Ecotoxicol Rev* (2013) 31(3):181-200. Epub 2013/09/13. doi: 10.1080/10590501.2013.824187. PubMed PMID: 24024518.

100. Tangutur NK, Medvedev SF, Regenstein F, Balart LA. Hepatocellular carcinoma, a rapidly increasing public health problem: the Tulane experience 2003-2009. *J La State Med Soc* (2011) 163(4):185-90. Epub 2011/10/01. PubMed PMID: 21954650.

101. Taura N, Yatsuhashi H, Nakao K, Ichikawa T, Ishibashi H. Long-term trends of the incidence of hepatocellular carcinoma in the Nagasaki prefecture, Japan. *Oncol Rep* (2009) 21(1):223-7. doi: 10.3892/or_00000212.

102. Torre LA, Islami F, Siegel RL, Ward EM, Jemal A. Global Cancer in Women: Burden and Trends. *Cancer Epidemiol Biomarkers Prev* (2017) 26(4):444-57. Epub 2017/02/23. doi: 10.1158/1055-9965.epi-16-0858. PubMed PMID: 28223433.

103. Tyson GL, Ilyas JA, Duan Z, Green LK, Younes M, El-Serag HB, et al. Secular Trends in the Incidence of Cholangiocarcinoma in the USA and the Impact of Misclassification. *Dig Dis Sci* (2014) 59(12):3103-10. doi: 10.1007/s10620-014-3276-2.

104. Utada M, Ohno Y, Shimizu S, Ito Y, Tsukuma H. Cancer incidence and mortality in Osaka, Japan: future trends estimation with an age-period-cohort model. *Asian Pac J Cancer Prev* (2012) 13(8):3893-8. Epub 2012/10/27. PubMed PMID: 23098489.

105. Valery PC, Laversanne M, Clark PJ, Petrick JL, McGlynn KA, Bray F. Projections of primary liver cancer to 2030 in 30 countries worldwide. *Hepatology* (2018) 67(2):600-11. doi: 10.1002/hep.29498.

106. Von Hahn T, Ciesek S, Wegener G, Plentz RR, Weismüller TJ, Wedemeyer H, et al. Epidemiological trends in incidence and mortality of hepatobiliary cancers in Germany. *Scand J Gastroenterol* (2011) 46(9):1092-8. doi: 10.3109/00365521.2011.589472.

107. Wang R, Chen XZ, Zhang MG, Tang L, Wu H. Incidence and mortality of liver cancer in mainland China: changes in first decade of 21st century. *Hepatogastroenterology* (2015) 62(137):118-21. Epub 2015/04/29. PubMed PMID: 25911880.

108. Waziry R, Grebely J, Amin J, Alavi M, Hajarizadeh B, George J, et al. Trends in hepatocellular carcinoma among people with HBV or HCV notification in Australia (2000–2014). *J Hepatol* (2016) 65(6):1086-93. doi: 10.1016/j.jhep.2016.08.010.

109. Weinmann A, Koch S, Niederle IM, Schulze-Bergkamen H, König J, Hoppe-Lotichius M, et al. Trends in epidemiology, treatment, and survival of hepatocellular carcinoma patients between 1998 and 2009: An analysis of 1066 cases of a german HCC registry. *J Clin Gastroenterol* (2014) 48(3):279-89. doi: 10.1097/MCG.0b013e3182a8a793.

110. Wilson RJ, Ryerson AB, Singh SD, King JB. Cancer incidence in appalachia, 2004-2011. *Cancer Epidemiol Biomarkers Prev* (2016) 25(2):250-8. doi: 10.1158/1055-9965.EPI-15-0946.

111. Wong IOL, Cowling BJ, Law SCK, Mang OWK, Schooling CM, Leung GM. Understanding sociohistorical imprint on cancer risk by age-period-cohort decomposition in Hong Kong. *J Epidemiol Community Health* (2010) 64(7):596-603. doi: 10.1136/jech.2008.080788. PubMed PMID: 105079811. Language: English. Entry Date: 20101001. Revision Date: 20150711. Publication Type: Journal Article.

112. Wong LL, Ogihara M, Ji J, Tsai N. The changing characteristics of hepatocellular cancer in Hawaii over time. *Am J Surg* (2015) 210(1):146-52. Epub 2014/12/17. doi: 10.1016/j.amjsurg.2014.06.036. PubMed PMID: 25510478.

113. Wu QJ, Vogtmann E, Zhang W, Xie L, Yang WS, Tan YT, et al. Cancer incidence among adolescents and young adults in urban Shanghai, 1973-2005. *PLoS One* (2012) 7(8). doi: 10.1371/journal.pone.0042607.

114. Yang B, Liu JB, So SK, Han SS, Wang SS, Hertz A, et al. Disparities in hepatocellular carcinoma incidence by race/ethnicity and geographic area in California: Implications for prevention. *Cancer* (2018) 124(17):3551-9. doi: 10.1002/cncr.31598. PubMed PMID: 30113700.

115. Yang JD, Ahmed Mohammed H, Harmsen WS, Enders F, Gores GJ, Roberts LR. Recent Trends in the Epidemiology of Hepatocellular Carcinoma in Olmsted County, Minnesota: A US Population-based Study. *J Clin Gastroenterol* (2017) 51(8):742-8. Epub 2017/04/27. doi: 10.1097/mcg.0000000000000810. PubMed PMID: 28445235; PubMed Central PMCID: PMCPMC5552490.

116. Yang JD, Kim B, Sanderson SO, St Sauver JL, Yawn BP, Pedersen RA, et al. Hepatocellular carcinoma in olmsted county, Minnesota, 1976-2008. *Mayo Clin Proc* (2012) 87(1):9-16. doi: 10.1016/j.mayocp.2011.07.001. PubMed PMID: 108218396. Language: English. Entry Date: 20120323. Revision Date: 20161117. Publication Type: journal article.

117. Yanik EL, Tamburro K, Eron JJ, Damania B, Napravnik S, Dittmer DP. Recent cancer incidence trends in an observational clinical cohort of HIV-infected patients in the US, 2000 to 2011. *Infect Agent Cancer* (2013) 8(1). doi: 10.1186/1750-9378-8-18.

118. Yépez MC, Jurado DM, Bravo LM, Bravo LE. Trends in cancer incidence, and mortality in pasto, Colombia. 15 years experience. *Colomb Med* (2018) 49(1):42-54. doi: 10.25100/cm.v49i1.3616.

119. Younossi ZM, Otgonsuren M, Henry L, Venkatesan C, Mishra A, Erario M, et al. Association of nonalcoholic fatty liver disease (NAFLD) with hepatocellular carcinoma (HCC) in the United States from 2004 to 2009. *Hepatology* (2015) 62(6):1723-30. doi: 10.1002/hep.28123. PubMed PMID: 26274335.

120. Zhang M, Higashi T. Time trends of liver cancer incidence (1973-2002) in Asia, from cancer incidence in five continents, Vols IV-IX. *Jpn J Clin Oncol* (2009) 39(4):275-6. Epub 2009/03/27. doi: 10.1093/jjco/hyp027. PubMed PMID: 19321855.

121. Zhang X, Condon JR, Rumbold AR, Cunningham J, Roder DM. Estimating cancer incidence in Indigenous Australians. *Aust N Z J Public Health* (2011) 35(5):477-85. Epub 2011/10/07. doi: 10.1111/j.1753-6405.2011.00762.x. PubMed PMID: 21973255.

122. Zhang Y, Ren JS, Shi JF, Li N, Wang YT, Qu C, et al. International trends in primary liver cancer incidence from 1973 to 2007. *BMC Cancer* (2015) 15:94. Epub 2015/04/17. doi: 10.1186/s12885-015-1113-4. PubMed PMID: 25879744; PubMed Central PMCID: PMCPMC4359785.

123. Zimmermann E, Berentzen TL, Gamborg M, Sørensen TIA, Baker JL. Sex-specific associations between birth weight and adult primary liver cancer in a large cohort of Danish children. *Int J Cancer* (2016) 138(6):1410-5. doi: 10.1002/ijc.29900.
