# Supplementary material for: Global Trends in Incidence Rates of Primary Adult Liver Cancers: A Systematic Review and Meta-Analysis
Source: Front Oncol. 2020 Feb 28;10:171. doi: 10.3389/fonc.2020.00171 (PMC7058661; doi:10.3389/fonc.2020.00171)
Supplement: Supplementary file 3 [file Table_3.PDF]

Supplementary file 3 Additional tables

1 Table S3.1 Quality checking for included studies

Author, year	Score[1]	Quality[2]
Bravo et al, 2018 (1)	10.5	Moderate
Carville et al, 2018 (2)	14	High
Chen et al, 2016 (3)	15	Moderate
Chinnaratha et al, 2016 (4)	15	High
Clark et al, 2015 (5)	16	High
Clèries et al, 2014 (6)	12	Moderate
Cocker et al, 2019 (7)	13	Moderate
Cooter et al, 2015 (8)	13.5	Moderate
Dryden-Peterson et al, 2015 (9)	13.5	Moderate
Gao et al, 2012 (10)	12.5	Moderate
Hung et al, 2015 (11)	14.5	High
Ito et al, 2011 (12)	12.5	Moderate
Jung et al, 2015 (13)	12.5	Moderate
Kamsa-ard et al, 2011 (14)	13	Moderate
Katanoda et al, 2015 (15)	13	Moderate
Kim et al, 2019 (16)	11	Moderate
Lepage et al, 2008 (17)	11.5	Moderate
Li et al, 2017 (18)	13.5	Moderate
Li et al, 2018 (19)	14	Moderate
Liu et al. 2019 (20)	13	Moderate
Lorenzoni et al, 2015 (21)	13	High
Medina et al, 2010 (22)	13	Moderate
Melkonian et al, 2018 (23)	14.5	High
Mutyaba et al, 2015 (24)	13	Moderate
Njei et al, 2015 (25)	15.5	High
Patel and Benipal, 2019 (26)	13	Moderate
Pham et al, 2018 (27)	12.5	Moderate
Pocobelli et al, 2008 (28)	13.5	Moderate
Polednak, 2013 (29)	13	Moderate
Ramirez et al, 2014 (30)	14	Moderate
Rich et al, 2019 (31)	14	High
Saha et al, 2016 (32)	14	High

Author, year	**Score[1]**	**Quality[2]**
Shamseddine et al, 2014 (33)	13	Moderate
Shiels et al, 2019 (34)	14	High
Shin et al, 2010 (35)	11.5	Moderate
Siegel et al, 2015 (36)	14	High
Sighoko et al, 2011 (37)	13.5	Moderate
Song et al, 2008 (38)	13.5	Moderate
Sung et al, 2019 (39)	14	High
Tanaka et al, 2008 (40)	13.5	Moderate
Thein et al, 2011 (41)	13	Moderate
Torre et al, 2016 (42)	12.5	Moderate
Van Dyke et al, 2019 (43)	14	High
Wallace et al, 2018 (44)	14	High
Wang et al, 2011 (45)	12	Moderate
Wang et al, 2019 (46)	15.5	High
Ward et al, 2019 (47)	14.5	High
White et al, 2017 (48)	15	High
Witjes et al, 2012a (49)	14	High
Witjes et al, 2012b (50)	13	High
Xu et al, 2017 (51)	14	High
Yeesoonsang et al, 2018 (52)	14	High
Zheng et al, 2018 (53)	11.5	Moderate

1. Average score over scores from two independent reviewers. Please refer to text for further details.
2. Quality categories: High (score14-18), Moderate (score 9-13.5) or Low (score <9); please refer to text for further details.

2 Table S3.2 Summary of included studies on trends in incidence rates for adult liver cancers combined

Author, year	Location	Period	Age-group (years)	Sex	Sample size	Period (trend)	APC (95% CI in brackets)[1,2,3]	Trend[4,5]
All included studies (arranged alphabetically by first author by country within continents; Africa, Americas, Asia, Europe and Oceania)								
Africa								
Dryden-Peterson et al, 2015 (9)	BOS	2003-2008	All	Persons	253	2003-2008	-6.6 (-12.0, -1.0)	Decreased
Sighoko et al, 2011 (37)	GAM	1988-2006	All	Males<br>Females	2,179<br>796	1988-2006	0.02 (-1.8, 1.9)<br>3.1 (0.3, 5.8)	Stable<br>Increased
Lorenzoni et al, 2015 (21)	Maputo, MOZ	1991-2008	All	Males<br>Females	NR	1991-2008	-1.6 (-4.0, 0.7)<br>2.4 (-1.1, 5.8)	Stable<br>Stable
Mutyaba et al, 2015 (24)	Kampala, UGA	1999-2008	All	Persons	481	1999-2008	7.2 (3.1, 11.5)	Increased
Americas								
Melkonian et al, 2018 (23)	USA[6]	1999-2009	All	Persons<br>Males<br>Females	28,592<br>20,011<br>8,581	1999-2009	NHW:3.6 (3.0, 4.3); AI/AN 5.0 (2.7, 7.3)<br>NHW:4.0 (3.3, 4.6); AI/AN 4.8 (2.0, 7.8)<br>NHW:2.2 (0.9, 3.6); AI/AN 5.2 (0.8, 9.9)	Increased (NHW, AI/AN)<br>Increased (NHW, AI/AN)<br>Increased (NHW, AI/AN)
Polednak, 2013 (29)	USA[6]	1999-2009	All	Persons	171,783	1999-2009	4.3 (4.1, 4.5)	Increased
Siegel et al, 2015 (36)	USA[7]	2003-2012	All	Males<br>Females	NR	2003-2012	1.8 (NHW), 3.7 (H) (95% CI NR)[8,9]<br>2.4 (NHW); 3.5 (H) (95% CI NR)[8,9]	Increased (NHW, H)<br>Increased (NHW, H)
Sung et al, 2019 (39)	USA[10,11]	1995-2014	25-29<br>30-34<br>35-39<br>40-44<br>45-49<br>50-54<br>55-59<br>60-64<br>65-69<br>70-74<br>75-79<br>80-84	Persons	NR	1995-2014	1.5 (-0.6, 3.6)<br>1.0 (-0.3, 2.4)<br>-0.2 (-1.2, 0.8)<br>-0.9 (-1.5, -0.2)<br>0.9 (0.5, 1.3)<br>5.3 (4.9, 5.6)<br>7.6 (7.3, 7.9)<br>6.0 (5.7, 6.3)<br>3.3 (3.0, 3.6)<br>2.5 (2.2, 2.8)<br>2.4 (2.1, 2.7)<br>2.0 (1.6, 2.5)	Stable<br>Stable<br>Stable<br>Decreased<br>Increased<br>Increased<br>Increased<br>Increased<br>Increased<br>Increased<br>Increased<br>Increased
Torre et al, 2016 (42)	USA[12]	2003-2012	All	Males<br>Females	NR	2003-2012	3.7 (NHW) (95% CI NR)[8,9]-1.7 (AANHPI)[8]<br>3.5 (NHW) (95% CI NR)[8,9], 1.3 (AANHPI)[8]	Increased (NHW), Stable (AANHPI)<br>Increased (NHW) , Stable (AANHPI)
Ward et al, 2019 (47)	USA[7]	1999-2015	All	Males<br>Females	NR	2011-2015	2.8 (2.1, 3.3)<br>3.8 (3.5, 4.0)	Increased<br>Increased
Bravo et al, 2018 (1)	COL	2008-2012	All	Males<br>Females	249<br>218	2008-2012	1.7 (0.9, 2.4)<br>0.4 (-0.5, 1.2)	Increased<br>Stable
Asia								
Chen et al, 2016 (3)	CHN	2000-2011	All	Males<br>Females	NR	2000-2011<br>2008-2011	-1.8 (95% CI NR)[8,9]<br>-4.4 (95% CI NR)[8,9]	Decreased<br>Decreased
Li et al, 2017 (18)	GZ, CHN	2004-2015	All	Persons<br>Males<br>Females	27,149<br>21,865<br>5,284	2004-2015	-2.3 (-3.5, -1.1)<br>-2.3 (-3.4, -1.1)<br>-2.3 (−4.1, −0.4)	Decreased<br>Decreased<br>Decreased
Li et al, 2018 (19)	SH, CHN	2002-2015	All	Persons<br>(Urban)	NR	2002-2015	-5.0 (-7.0, -3.1)	Decreased

Author, year	Location	Period	Age-group (years)	Sex	Sample size	Period (trend)	APC (95% CI in brackets)[1,2,3]	Trend[4,5]
Liu et al. 2019 (20)	SH, CHN	1988-2013	All	Males<br>Females	NR	1988-2013	-1.9 (95% CI NR)[8,9]<br>-2.3 (95% CI NR)[8,9]	Decreased<br>Decreased
Song et al, 2008 (38)	TJ, CHN	1981-2000	All	Males<br>Females	NR	1981-2000	-1.1 (-1.6, -0.7)<br>-1.2 (-1.9, -0.3)	Decreased<br>Decreased
Wang et al, 2011 (45)	BJ, CHN	1998-2007	All	Males<br>Females	8,820<br>3,082	1998-2007	0.4 (95% CI NR)[7]<br>-0.8 (95% CI NR)[7]	Stable<br>Stable
Xu et al, 2017 (51)	SZ, CHN	2001-2015	All	Males<br>Females	NR	2001-2015	-1.1 (95% CI NR)[7]<br>-1.7 (95% CI NR)[7]	Stable<br>Stable
Zheng et al, 2018 (53)	CHN	2000-2014	All	Persons	NR	2000-2014	-2.3 (95% CI NR) [8,9]	Decreased
Ito et al, 2011 (12)	Osaka, JPN	1968-2007	All	Persons<br>Males<br>Females	NR	1998-2007	-3.7 (95% CI NR)[8,9]<br>-4.3 (95% CI NR)[8,9]<br>-2.1 (95% CI NR)[8,9]	Decreased<br>Decreased<br>Decreased
Katanoda et al, 2015 (15)	Yamagata, Fukui & Nagasaki, JPN	1985-2010	All	Males<br>Females	NR	1992-2010<br>1995-2010	-2.2 (-2.7, -1.8)<br>-1.5 (-2.2, -0.9)	Decreased<br>Decreased
Jung et al, 2015 (13)	KOR	1999-2012	All	Persons<br>Males<br>Females	NR	1999-2012	-1.9 (95% CI NR)[8,9]<br>-2.1 (95% CI NR)[8,9]<br>-1.6 (95% CI NR)[8,9]	Decreased<br>Decreased<br>Decreased
Shamseddine et al, 2014 (33)	LEB	2003-2008	All	Males<br>Females	NR	2003-2008	13.6 (95% CI NR)[8,9]<br>18.3 (95% CI NR)[8,9]	Increased<br>Increased
Medina et al, 2010 (22)	PHL	1983-2002	<75	Males<br>Females	4,177<br>1,538	1983-2002	-1.0 (-1.7, -0.3)<br>-1.2 (-2.1, -0.3)	Decreased<br>Decreased
Europe								
Cooter et al, 2015 (8)	CYP	1998-2008	All	Males<br>Females	80<br>35	1998-2008	4.4 (1.3, 8.8)<br>8.3 (1.5, 15.5)	Increased<br>Increased
Lepage et al, 2008 (17)	FRA	1980-2000	All	Males<br>Females	NR	1980-2000	4.8 (95% CI NR)[8,9]<br>3.4 (95% CI NR)[8,9]	Increased<br>Increased
Witjes et al, 2012a (49)	NLD	1989-2009	All	Males<br>Females	4,415<br>2,099	1989-2009	2.1 (1.5, 2.7)<br>1.0 (0.2, 1.8)	Increased<br>Increased
Clèries et al, 2014 (6)	ESP	1993-2007	All	Males<br>Females	560<br>229	1993-2007	2.2 (95% CI NR)[8]<br>-0.6 (95% CI NR)[8]	Stable<br>Stable
Oceania								
Cocker et al, 2019 (7)	AUS	1982-2014	All	Persons<br>Males<br>Females	NR	2006-2014<br>1982-2014<br>2005-2014	3.7 (2.9, 4.5)<br>4.6 (4.4, 4.8)<br>3.0 (4.6, 5.6)	Increased<br>Increased<br>Increased
Additional trends by age-								
Polednak, 2013 (29)	USA[6]	1999-2009	15-34<br>35-44<br>45-54<br>55-64	Persons	2,049<br>5,714<br>34,461<br>45,145	1999-2009	2.0 (–0.2, 4.2)<br>-0.9 (–1.5, -0.3)<br>5.0 (3.6, 6.4)<br>8.3 (7.3, 9.3)	Stable<br>Decreased<br>Increased<br>Increased

Author, year	Location	Period	Age-group (years)	Sex	Sample size	Period (trend)	APC (95% CI in brackets)[1,2,3]	Trend[4,5]
			65-74<br>75-84<br>85+		40,643<br>33,668<br>10,103		2.8 (2.4, 3.2)<br>3.0 (2.6, 3.4)<br>1.2 (0.8, 1.6)	Increased<br>Increased<br>Increased
Li et al, 2017 (18)	CHN	2004-2015	0-34<br>35-54<br>55-74<br>75+	Persons	NR	2004-2015	-6.0 (-8.0, -4.0)<br>-2.1 (-3.3, -0.8)<br>-2.5 (-3.8, -1.1)<br>-0.2 (-2.8, 2.6)	Decreased<br>Decreased<br>Decreased<br>Stable
Zheng et al, 2018 (53)	CHN	2000-2014	<40<br>40-49<br>50-59<br>60-69<br>70-79<br>80+	Persons	NR	2000-2014	-3.9 (95% CI NR)[8,9]<br>-3.0 (95% CI NR)[8,9]<br>-1.6 (95% CI NR)[8,9]<br>-2.2 (95% CI NR)[8,9]<br>-1.8 (95% CI NR)[8,9]<br>-0.6 (95% CI NR)[8]	Decreased<br>Decreased<br>Decreased<br>Decreased<br>Decreased<br>Stable

AANHPI Asian American, Native Hawaiians & Pacific Islanders; AI/AN American Indians/Alaska Native; APC Annual Percentage Change; AUS Australia; BOS Botswana; BJ Beijing; CHN China; COL Colombia; CYP Cyprus; ESP Spain; FRA France; GAM Gambia; GZ Guangzhou; H Hispanic; JPN Japan; KOR South Korea; LEB Lebanon; MOZ Mozambique; NLD Netherlands; NR Not reported, NHW Non-Hispanic white; PHL Philippines; SH, Shanghai, SZ Shenzhen; TJ Tianjin; UGA Uganda; USA United States

1. Adult liver cancers were defined according to the International Classification of Disease for Oncology, Third Edition (ICD-O-3) or International Classification of Disease, Tenth Edition (ICD-10) site code (C22).
2. Annual percentage change in age-standardized rates determined using JoinPoint regression except for four studies (8, 9, 21, 24) that used Poisson modelling and two studies (17, 39) that used age-period-cohort methods.
3. Trends based on annual percentage change (APC) in age-standardised incidence rate. The APC is the annual increase or decrease in incidence trends over the specified time period.
4. Negative APC values indicate a decreasing trend whereas positive APC values indicate an increasing trend. Stable means that the 95% confidence interval does not include zero.
5. Only incidence trends for most recent time period shown.
6. Based on population-based cancer incidence data from the United States Cancer Statistics registry for all 50 states in the USA and the District of Colombia.
7. Based on population-based cancer incidence data from the North American Association of Central Cancer Registries database for 47 states and the District of Puerto Rico in the USA.
8. Studies did not report the 95% confidence intervals and were not included in the meta-analysis.
9. Findings were significant at 5% level.
10. Based on population-based cancer incidence data from the North American Association of Central Cancer Registries database for 25 states in the USA.
11. Study not included in meta-analysis as trends only reported by smaller age-groups.
12. Based on population-based cancer incidence data from the North American Association of Central Cancer Registries database for 24 states and one metropolitan area (Atlanta) in the USA.

3 Table S3.3 Summary of included studies on trends in incidence rates for adult hepatocellular carcinoma

Author, year	Location	Period	Age-group (years)	Sex	Sample size	Period (trend)	APC (95% CI in brackets)[1,2,3]	Trend[4,5]
All included studies (arranged alphabetically by first author by country within continents; Americas, Asia, Europe and Oceania)								
**Americas**								
Njei et al, 2015 (25)	USA[6]	1973-2010	All	Persons<br>Males<br>Females	63,297<br>47,346<br>15,951	2007-2011<br>2006-2011<br>2009-2011	2.7 (0.6, 4.9)<br>3.5 (95% CI NR)[7]<br>-2.2 (95% CI NR)	Increased<br>Increased<br>Stable
Pham et al, 2018 (27)	CA, USA	1988-2012	All	Males (NHW)<br>Females (NHW)	12,807<br>4,023	1988-2012<br>1988-2012	5.3 (4.9, 5.6)<br>3.8 (3.3, 4.3)	Increased<br>Increased
Ramirez et al, 2014 (30)	TX, USA	1995-2010	All	Persons (NHW)	13,584	1995-2010	5.5 (4.8, 6.2)	Increased
Rich et al, 2019 (31)	USA[8,9]	1992-2015	20+	Persons	51,888	2010-2015	-0.7 (-2.0, 0.7)	Stable
Shiels et al, 2019 (34)	USA[10,11]	2001-2013	66-99	Persons<br>Males<br>Females	15,300<br>10,262<br>5,038	2001-2013	3.4 (2.8, 4.0)<br>3.2 (2.5, 3.8)<br>3.1 (2.2, 4.1)	Increased<br>Increased<br>Increased
White et al, 2017 (48)	USA[12]	2000-2012	All	Persons<br>Males<br>Females	236,290<br>172,492<br>63,798	2000-2012	3.5 (3.3, 3.8)<br>3.7 (3.3, 4)<br>2.7 (2.2, 3.2)	Increased<br>Increased<br>Increased
Pocobelli et al, 2008 (28)	CAN	1976-2000	20+	Males<br>Females	NR	1976-2000	3.4 (3.0, 3.8)<br>2.2 (1.5, 2.8)	Increased<br>Increased
**Asia**								
Gao et al, 2012 (10)	SH, CHN	1975-2005	25-79	Males<br>Females	35,241<br>13,931	1975-2005	-1.6 (-1.8, -1.4)<br>-1.8 (-2.0, 1.6)	Decreased<br>Stable
Tanaka et al, 2008 (40)	Osaka, JPN[11]	1981-2003	50-59<br>60-69<br>70-79<br>50-59<br>60-69<br>70-79	Males<br><br><br>Females	NR	1996-2003<br>1995-2003<br>2000-2003<br>1991-2003<br>1997-2003<br>2000-2003	-3.1 (-4.2, -2.1)<br>-22.3 (-26, -18.6)<br>-12.4 (-35.7, 10.9)<br>-0.9 (-1.1, -0.7)<br>-5.7 (-7.3, -4.1)<br>-7.9 (-18.1, 2.4)	Decreased<br>Decreased<br>Stable<br>Decreased<br>Decreased<br>Stable
Yeesoonsang et al, 2018 (52)	Khon-Kaen, THA	1989-2009<br>1989-2013	20+	Males<br>Females	1,004<br>184	2007-2013<br>1989-2013	-2.8 (-9.8, 4.8)<br>1.4 (0.1, 2.7)	Stable<br>Increased
Hung et al, 2015 (11)	TWN	2003-2011	All	Persons<br>Males<br>Females	82,856<br>60,117<br>22,739	2003-2011	-0.5 (-1.2, 0.2)<br>-0.4 (-1.1, 0.3)<br>-0.2 (-1.1, 0.6)	Stable<br>Stable<br>Stable
**Europe**								
Witjes et al, 2012a (49)	NLD	1982-2014	15-95	Persons<br>Males<br>Females	5,143<br>3,738<br>1,405	1989-2009	0.6 (-0.1, 1.3)<br>2.2 (1.6, 2.7)<br>1.0 (-0.01, 2.2)	Stable<br>Increased<br>Stable

Author, year	Location	Period	Age-group (years)	Sex	Sample size	Period (trend)	APC (95% CI in brackets)[1,2,3]	Trend[4,5]
**Oceania**								
Carville et al, 2018 (2)	VIC, AUS	2004-2013	All	Males<br>Females	1,737<br>434	2004-2013	8.6 (6.3, 10.9)<br>8.3 (4.7, 11.9)	Increased<br>Increased
Chinnaratha et al, 2016 (4)	SA, AUS[11]	1996-2010	All	Persons (HBV+)[13]	47	1996-2010	20.8 (10.1, 32.5)	Increased
Clark et al, 2015 (5)	QLD, AUS	1996-2011	All	Males<br>Females	1,315<br>305	1996-2011	3.5 (2.1, 5.0)<br>2.6 (-0.7, 6.0)	Increased<br>Stable
Thein et al, 2011 (41)	NSW, AUS[10]	1992-2007	All	Persons (HBV+)[13]<br>Persons (HCV+)[13]	329<br>446	1992-2007	-3.3 (-8.2, 1.8)<br>-3.3 (-6.3, -0.2)	Stable<br>Decreased
Wallace et al, 2018 (44)	AUS	1982-2013	45+	Persons<br>Males<br>Females	18,575<br>14,777<br>3,645	1982-2014	4.5 (4.2, 4.7)<br>4.3 (4.1, 4.6)<br>4.2 (3.8, 4.6)	Increased<br>Increased<br>Increased
**Additional trends by age**								
Ramirez et al, 2014 (30)	TX, USA	1995-2010	50-59<br>60-69<br>70-79<br>80+	Persons (NHW)	NR	1995-2010	12.4 (10.9, 13.9)<br>4.7 (3.1, 6.3)<br>2.7 (1.4, 4.1)<br>2.7 (1.5, 4.0)	Increased<br>Increased<br>Increased<br>Increased
Rich et al, 2019 (31)	USA[8]	1992-2015	40-49<br>50-59<br>60-69<br>70-79<br>80+	Persons (NHW)	NR	2009-2015<br>2012-2015<br>2004-2015<br>1992-2015<br>1992-2015	-12.2 (-18.9, -5.1)<br>-9.0 (-15.8, -1.6)<br>8.7 (7.7, 9.7)<br>2.6 (2.2, 3.0)<br>3.2 (2.6, 3.8)	Decreased<br>Decreased<br>Increased<br>Increased<br>Increased
Shiels et al, 2019 (34)	USA[10]	2001-2013	66-75<br>76-85<br>86+	Persons	8,006<br>6,039<br>1,255	2001-2013	3.2 (2.4, 4.0)<br>3.9 (3.2, 4.6)<br>2.8 (0.9, 4.7)	Increased<br>Increased<br>Increased
White et al, 2017 (48)	USA[12]	2000-2012	20-24<br>25-29<br>30-34<br>35-39<br>40-44<br>45-49<br>50-54<br>55-59<br>60-64<br>65-69<br>70-74<br>75-79<br>80-84<br>85+	Persons	NR	2000-2012	1.0 (-2.8, 5.0)<br>0.0 (-8.8, 9.6)<br>1.5 (-0.9, 4.1)<br>-2.7 (-4.4, -1.0)<br>-2.0 (-3.0, -1.0)<br>-1.6 (-3.2, 0.0)<br>4.4 (2.5, 6.2)<br>8.9 (7.1, 10.7)<br>6.4 (4.7, 8.2)<br>3.1 (2.7, 3.5)<br>2.2 (1.6, 2.8)<br>2.1 (1.3, 3.0)<br>2.6 (1.8, 3.5)<br>1.8 (1.3, 2.3)	Stable<br>Stable<br>Stable<br>Decreased<br>Decreased<br>Decreased<br>Increased<br>Increased<br>Increased<br>Increased<br>Increased<br>Increased<br>Increased<br>Increased
Hung et al, 2015 (11)	TWN	2003-2011	15-29<br>30-64<br>65+	Persons	624<br>41,503<br>40,692	2000-2003	-7.9 (-10.0, -5.7)<br>-2.0 (-2.8, -1.1)<br>1.3 (0.6, 1.9)	Decreased<br>Decreased<br>Increased

Author, year	Location	Period	Age-group (years)	Sex	Sample size	Period (trend)	APC (95% CI in brackets)[1,2,3]	Trend[4,5]
Wallace et al, 2018 (44)	AUS	1982-2014	45-49	Persons	992	1982-2014	2.0 (-2.4, 6.6)	Stable
			50-54		1,808		4.8 (2.8, 6.8)	Increased
			55-59		2,218		4.9 (6.2, 7.4)	Increased
			60-64		2,410		4.1 (2.7, 5.6)	Increased
			65-69		2,706		3.1 (2.3, 4.0)	Increased
			70-74		2,764		4.7 (3.5, 5.9)	Increased
			75-79		2,371		4.3 (3.1, 5.6)	Increased
			80-84		1,495		5.4 (4.6, 6.2)	Increased
			85+		855		4.7 (2.9, 6.5)	Increased
**Additional trends by sex & age-group**								
Pham et al, 2018 (27)	CA, USA	1988-2012	20-39	Males (NHW)	177	1988-2012	1.4 (-0.4, 3.3)	Stable
			40-49		981		5.1 (2.6, 7.6)	Increased
			50-59		3,776		9.8 (8.8, 10.9)	Increased
			60-69		3,702		5.3 (4.5, 6.1)	Increased
			70+		4,171		3.0 (2.7, 3.4)	Increased
			20-39	Females (NHW)	104		-0.1 (-2.7, 2.6)	Stable
			40-49		236		2.2 (0.4, 3.9)	Increased
			50-59		693		6.0 (4.3, 7.8)	Increased
			60-69		980		3.9 (2.6, 5.2)	Increased
			70+		2,010		3.5 (2.8, 4.3)	Increased
Pocobelli et al, 2008 (28)	CAN	1976-2000	20-49	Males	NR	1976-2000	3.3 (2.3, 4.3)	Increased
			50-64				3.3 (2.7, 3.9)	Increased
			65-74				3.7 (3.2, 4.3)	Increased
			74+				2.6 (2.1, 3.1)	Increased
			20-49	Females			1.1 (-0.01, 2.3)	Stable
			50-64				2.2 (1.3, 3.0)	Increased
			65-74				3.1 (2.1, 4.1)	Increased
			74+				1.5 (0.7, 4.3)	Increased
Witjes et al, 2012a (49)	NLD	1989-2009	<60	Males	1,064	1989-2009	2.9 (1.6, 3.9)	Increased
			60-74		1,763		1.7 (0.8, 2.5)	Increased
			75+		911		2.6 (1.3, 3.8)	Increased
			<60	Females	352		2.6 (0.3, 5.0)	Increased
			60-74		566		0.2 (-1.6, 2.1)	Stable
			75+		487		0.9 (-0.4, 2.3)	Stable
Clark et al, 2015 (5)	QLD, AUS	1996-2011	<50	Males	NR	1996-2011	3.9 (0.4, 7.6)	Increased
			50-69				4.2 (2.0, 6.5)	Increased
			70+				2.1 (0.4, 3.9)	Increased
			<50	Females			2.1 (-6.8, 11.9)	Stable
			50-69				-1.4 (-3.4, 6.5)	Stable
			70+				3.6 (-0.6, 8.0)	Stable

APC Annual Percentage Change; API Asian/Pacific Islander; AUS Australia; CA California; CAN Canada; CHN China; H Hispanic; HBV Hepatitis B virus; HCV Hepatitis C virus; JPN Japan; NLD Netherlands; NR Not reported, NHW Non-Hispanic white; NSW New South Wales; QLD Queensland; SA South Australia; SH Shanghai; THA Thailand; TWN Taiwan; TX Texas; USA United States; VIC Victoria

1. Adult hepatocellular carcinomas (HCC) were defined according to the International Classification of Disease for Oncology, Third Edition (ICD-O-3) site code (C22.0) and histologically as HCC (ICD-O-3 morphology codes M8170-M8175).
2. Annual percentage change in age-standardized rates determined using JoinPoint regression except for three studies (2, 10, 41) that used Poisson modelling and one study (28) that used age-period-cohort methods.
3. Trends based on annual percentage change (APC) in age-standardised incidence rate. The APC is the annual increase or decrease in incidence trends over the specified time period.
4. Negative APC values indicate a decreasing trend whereas positive APC values indicate an increasing trend. Stable means that the 95% confidence interval does not include zero.
5. Only incidence trends for most recent time period shown.
6. Based on population-based cancer incidence data from the Surveillance, Epidemiology and End Results (SEER 18) database covering 11 states and two metropolitan areas in the USA.
7. Findings were significant at 5% level.
8. Based on population-based cancer incidence data from the Surveillance, Epidemiology and End Results (SEER 13) database covering six states and seven regions in the USA.
9. Also gives trends by smaller age group and ethnicity.
10. Based on data from the Surveillance, Epidemiology and End Results/Medicare linked database.
11. Not included in meta-analysis as trends only reported for population sub-groups.
12. Based on population-based cancer incidence data from the United States Cancer Statistics registry for all 50 states and the District of Colombia in the USA.
13. Only people infected with viral hepatitis B (HBV+) or viral hepatitis C (HCV+).

4 Table S3.4 Summary of included studies on trends in incidence rates for intrahepatic cholangiocarcinoma or combined hepatocellular and cholangiocarcinoma

Author, year	Location	Period	Age group (years)	Sex	Sample size	Period (trend)	APC (95% CI in brackets)[1,2,3]	Trend[4,5]
**Asia**								
**Intrahepatic Cholangiocarcinoma[1]**								
**Americas**								
Patel and Benipal, 2019 (26)	USA[6]	2001-2015	0-99	Persons	NR	2010-2015	9.7 (95% CI NR)[7,8]	Increased
Saha et al, 2016 (32)	USA[9]	1973-2012	0-99	Persons	NR	2003-2012	4.4 (3.4, 5.3)	Increased
Van Dyke et al, 2019 (43)	USA[10]	1999-2013	15+	Persons<br>Males<br>Females	37,685<br>19,438<br>18,247	1999-2013	3.2 (95% CI NR)[7,8]<br>2.6 (95% CI NR)[7,8]<br>3.7 (95% CI NR)[7,8]	Increased<br>Increased<br>Increased
**Asia**								
Kamsa-ard et al, 2011 (14)	Songkhla, THA[11]	1985-2009	15+	Persons<br>Males<br>Females	10,730<br>7,419<br>3,311	2003-2009<br>2002-2009<br>2003-2009	-7.5 (-13.5, -1.0)<br>-5.8 (-10.4, -1.0)<br>-8.1 (-14.6, -1.1)	Decreased<br>Decreased<br>Decreased
Kim et al, 2019 (16)	KOR	2006-2015	20+	Persons	34,695	2006-2015	-1.3 (95% CI NR)[7,8]	Decreased
Shin et al, 2010 (35)	KOR	1999-2005	30-89	Males<br>Females	6,489<br>3,834	1999-2005	7.9 (95% CI NR)[7,8]<br>10.6 (95% CI NR)[7,8]	Increased<br>Increased
Yeesoonsang et al, 2018 (52)	Khon-Kaen, THA[11]	1989-2013	20+	Males<br>Females	657<br>363	1989-2013	5.2 (3.8, 6.6)<br>4.4 (3.1, 5.7)	Increased<br>Increased
**Europe**								
Witjes et al, 2012b (50)	NLD	1989-2009	15-95	Persons	785	1999-2009	9.4 (4.6, 14.3)	Increased
**Additional trends by age**								
Van Dyke et al, 2019 (43)	USA[10]	1999-2013	<45<br>45+	Persons	NR	1999-2013	3.3 (95% CI NR)[7,8]<br>3.2 (95% CI NR)[7,8]	Increased<br>Increased
Witjes et al, 2012b (50)	NLD	1989-2009	30-44<br>45-59<br>60-74<br>75+	Persons	NR	1989-2009	2.7 (-2.6, 7.9)<br>3.0 (0.2, 5.8)<br>0.7 (-2.1, 3.5)<br>1.8 (-0.7, 4.3)	Stable<br>Increased<br>Stable<br>Stable
**Combined Hepatocellular and Cholangiocarcinoma[1]**								
Wang et al, 2019 (46)	USA[9]	2000-2014	0-99	Persons<br>Males<br>Females	642<br>433<br>209	2000-2014	5.7 (1.6, 10.0)<br>NR	Increased<br>Increased<br>NR

APC Annual Percentage Change; KOR Korea; NLD Netherlands; NR Not reported; THA Thailand; USA United States of America

1. Adult liver cancers were defined according to the International Classification of Disease for Oncology, Third Edition (ICD-O-3) or International Classification of Disease, Tenth Edition (ICD-10) site code (C22) and histologically as cholangiocarcinoma (CHCA, ICD-O-3 site codes C22.1, C24.0), intrahepatic cholangiocarcinoma (ICC, ICD-O-3 site code C22.1, morphology code M8160) or combined hepatocellular-cholangiocarcinoma (cHCC-CC, ICD-O-3 morphology code M8180).
2. Annual percentage change in age-standardized rates determined using JoinPoint regression.
3. Trends based on annual percentage change (APC) in age-standardised incidence rate. The APC is the annual increase or decrease in incidence trends over the specified time period.
4. Negative APC values indicate a decreasing trend whereas positive APC values indicate an increasing trend. Stable means that the 95% confidence interval does not include zero.
5. Only incidence rates and trends for most recent time period shown.
6. Based on data from the United States Cancer Statistics registry which includes population-based cancer incidence data for all 50 states in the United States and the District of Colombia.
7. Studies did not report the 95% confidence intervals and were not included in the meta-analysis.
8. Findings were significant at 5% level.
9. Based on population-based cancer incidence data from the Surveillance, Epidemiology and End Results (SEER 18) database covering 11 states and two metropolitan areas in the USA.
10. Based on population-based cancer incidence data from the North American Association of Central Cancer Registries database for 38 states in the USA.
11. Studies by Kamsa-ard et al, 2011 (14) and Yeesoonsang et al, 2018 (52) includes all cholangiocarcinoma cases (CHCA, ICD-O-3 site codes C22.1, C24.0).

Table S3.5 Summary of most recent liver cancer incidence trends for included studies from United States that present additional trend estimates by race/ethnicity

Author, year	Time-period	Sex	Age group (years)[1]	Trends[2,3,4,5] by Race/Ethnicity[6,7] White	NHW	Black	API	AIAN	NHAI/AN	Hispanic	non-Hispanic
				<u>Liver cancers combined[8]</u>							
Melkonian et al, 2018 (23)	1999-2009	Persons	All	NR	↑	NR	NR	NR	↑	NR	NR
Siegel et al, 2015 (36)	2003-2012	Males	All	NR	↑	NR	NR	NR	NR	↑	NR
Siegel et al, 2015 (36)	2003-2012	Females	All	NR	↑	NR	NR	NR	NR	↑	NR
Torre et al, 2016 (42)	2003-2012	Males	All	NR	↑	NR	↓	NR	NR	NR	NR
Torre et al, 2016 (42)	2003-2012	Females	All	NR	↑	NR	↔	NR	NR	NR	NR
Ward et al, 2019 (47)	2011-2015	Males	All	↑	NR	↑	↓	↑	NR	↔	↑
Ward et al, 2019 (47)	2011-2015	Females	All	↑	NR	↑	↔	↑	NR	↑	↑
				<u>Hepatocellular carcinoma[8]</u>							
Pham et al, 2018 (27)	1988-2012	Males	All	NR	↑	↑	↑	NR	NR	↑	NR
Pham et al, 2018 (27)	1988-2012	Females	All	NR	↑	↑	↑	NR	NR	↑	NR
Ramirez et al, 2014 (30)	1995-2010	Persons	All	NR	↑	NR	NR	NR	NR	↑	NR
Shiels et al, 2019 (34)	2001-2013	Persons	All	↑	NR	↑	↔	NR	NR	↑	NR
White et al, 2017 (48)	2000-2012	Persons	All	↑	NR	↑	↓	↑	NR	↑	NR
Rich et al, 2019 (31)	2009-2015	Persons	30-39	NR	NS	NS	↓	NR	NR	↔	NR
Rich et al, 2019 (31)	2009-2015	Persons	40-59	NR	↓	↓	↓	NR	NR	↓	NR
Rich et al, 2019 (31)	2004-2015	Persons	60-69	NR	↑	↑	↔	NR	NR	↑	NR
Rich et al, 2019 (31)	1992-2015	Persons	70-79	NR	↑	↑	↓	NR	NR	↑	NR
				<u>Intrahepatic cholangiocarcinoma[8]</u>							
Van Dyke et al, 2019 (43)	1999-2013	Persons	15+	NR	↑	↑	NR	NR	NR	↑	NR

API Asian/Pacific Islander; AI/AN American Indian/Alaska Native; H Hispanic; NHAI/AN non-Hispanic American Indian/Alaska Native; NHW non-Hispanic White; NR not reported; NS not stated;

1. Only age groups for which incidence trends were reported are presented. Hence for some studies, only certain age groups are shown.
2. Trends based on annual percentage change (APC) in age-standardised incidence rate. The APC is the annual increase or decrease in incidence trends over the specified time period.
3. Negative APC values indicate a decreasing trend whereas positive APC values indicate an increasing trend. Stable means that the 95% confidence interval does not include zero.
4. Increasing trends indicated by red arrow; Decreasing by green arrow and stable trends by blue arrow.
5. Only incidence trends for most recent time period shown.
6. Race categories are not mutually exclusive from Hispanic origin unless specially stated as non-Hispanic white, non-Hispanic Asian/Pacific Islander or non-Hispanic American Indian/Alaska Native
7. Data for specified racial or ethnic populations other than whites and Blacks should be interpreted cautiously.
8. Adult liver cancers were defined according to the International Classification of Disease for Oncology, Third Edition (ICD-O-3) or International Classification of Disease, Tenth Edition (ICD-10) site code (C22) and histologically as hepatocellular carcinoma (HCC) (ICD-O-3 site code C22.0, morphology codes M8170-M8175) or intrahepatic cholangiocarcinoma (ICC, C22.1, M8170).

6 References

1. Bravo LE, García LS, Collazos P, Carrascal E, Ramírez O, Collazos T, et al. Reliable information for cancer control in Cali, Colombia. *Colomb Med* (2018) 49(1):23-34. doi: 10.25100/cm.v49i1.3689.

2. Carville KS, MacLachlan JH, Thursfield V, Cowie BC. Hepatocellular carcinoma over three decades in Victoria, Australia: epidemiology, diagnosis and trends, 1984–2013. *Intern Med J* (2018) 48(7):835-44. doi: 10.1111/imj.13823.

3. Chen W, Zheng R, Baade PD, Zhang S, Zeng H, Bray F, et al. Cancer statistics in China, 2015. *CA Cancer J Clin* (2016) 66(2):115-32. Epub 2016/01/26. doi: 10.3322/caac.21338. PubMed PMID: 26808342.

4. Chinnaratha MA, Graham C, Fraser RJL, Woodman RJ, Wigg AJ. Rising incidence of hepatitis B-related hepatocellular carcinoma in South Australia: 1996–2010. *Intern Med J* (2016) 46(8):902-8. doi: 10.1111/imj.13121.

5. Clark PJ, Stuart KA, Leggett BA, Crawford DH, Boyd P, Fawcett J, et al. Remoteness, race and social disadvantage: disparities in hepatocellular carcinoma incidence and survival in Queensland, Australia. *Liver Int* (2015) 35(12):2584-94. doi: 10.1111/liv.12853. PubMed PMID: 25900432.

6. Clèries R, Esteban L, Borràs J, Marcos-Gragera R, Freitas A, Carulla M, et al. Time trends of cancer incidence and mortality in Catalonia during 1993-2007. *Clinical and Translational Oncology* (2014) 16(1):18-28. doi: 10.1007/s12094-013-1060-y.

7. Cocker F, Chien Yee K, Palmer AJ, de Graaff B. Increasing incidence and mortality related to liver cancer in Australia: time to turn the tide. *Aust N Z J Public Health* (2019) 43(3):267-73. doi: 10.1111/1753-6405.12889. PubMed PMID: 30958629.

8. Cooter M, Soliman AS, Pavlou P, Demetriou A, Orphanides C, Kritioti E, et al. Incidence and time trends of cancer in Cyprus over 11 years (1998-2008). *Tumori* (2015) 101(1):8-15. doi: 10.5301/tj.5000204.

9. Dryden-Peterson S, Medhin H, Kebabonye-Pusoentsi M, Seage GR, Suneja G, Kayembe MKA, et al. Cancer incidence following expansion of HIV treatment in Botswana. *PLoS One* (2015) 10(8). doi: 10.1371/journal.pone.0135602.

10. Gao S, Yang WS, Bray F, Va P, Zhang W, Gao J, et al. Declining rates of hepatocellular carcinoma in urban Shanghai: Incidence trends in 1976-2005. *Eur J Epidemiol* (2012) 27(1):39-46. doi: 10.1007/s10654-011-9636-8.

11. Hung GY, Horng JL, Yen HJ, Lee CY, Lin LY. Changing incidence patterns of hepatocellular carcinoma among age groups in Taiwan. *J Hepatol* (2015) 63(6):1390-6. doi: 10.1016/j.jhep.2015.07.032. PubMed PMID: 26256438.

12. Ito Y, Ioka A, Nakayama T, Tsukuma H, Nakamura T. Comparison of trends in cancer incidence and mortality in Osaka, Japan, using an age-period-cohort model. *Asian Pac J Cancer Prev* (2011) 12(4):879-88. Epub 2011/07/28. PubMed PMID: 21790220.

13. Jung KW, Won YJ, Kong HJ, Oh CM, Cho H, Lee DH, et al. Cancer statistics in Korea: Incidence, mortality, survival, and prevalence in 2012. *Cancer Res Treat* (2015) 47(2):127-41. doi: 10.4143/crt.2015.060.

14. Kamsa-ard S, Wiangnon S, Suwanrungruang K, Promthet S, Khuntikeo N, Kamsa-ard S, et al. Trends in liver cancer incidence between 1985 and 2009, Khon Kaen, Thailand: cholangiocarcinoma. *Asian Pac J Cancer Prev* (2011) 12(9):2209-13.

15. Katanoda K, Hori M, Matsuda T, Shibata A, Nishino Y, Hattori M, et al. An updated report on the trends in cancer incidence and mortality in Japan, 1958-2013. *Jpn J Clin Oncol* (2015) 45(4):390-401. doi: 10.1093/jjco/hyv002.

16. Kim BW, Oh CM, Choi HY, Park JW, Cho H, Ki M. Incidence and Overall Survival of Biliary Tract Cancers in South Korea from 2006 to 2015: Using the National Health Information Database. *Gut and Liver* (2019) 13(1):104-13. doi: 10.5009/gnl18105. PubMed PMID: WOS:000455765000017.

17. Lepage C, Remontet L, Launoy G, Trétarre B, Grosclaude P, Colonna M, et al. Trends in incidence of digestive cancers in France. *Eur J Cancer Prev* (2008) 17(1):13-7.

18. Li K, Lin G, Li Y, Xu H. Time trends and patterns of primary liver cancer in Guangzhou from 2004 to 2015. *Precision Radiation Oncology* (2017) 1(4):116-20. doi: 10.1002/pro6.30.

19. Li X, Deng Y, Tang W, Sun Q, Chen Y, Yang C, et al. Urban-Rural Disparity in Cancer Incidence, Mortality, and Survivals in Shanghai, China, During 2002 and 2015. *Front Oncol* (2018) 8:579. doi: 10.3389/fonc.2018.00579. PubMed PMID: 30560091; PubMed Central PMCID: PMCPMC6287035.

20. Liu Z, Jiang Y, Fang Q, Yuan H, Cai N, Suo C, et al. Future of cancer incidence in Shanghai, China: Predicting the burden upon the ageing population. *Cancer Epidemiol* (2019) 60:8-15. doi: 10.1016/j.canep.2019.03.004.

21. Lorenzoni C, Vilajeliu A, Carrilho C, Ismail MR, Castillo P, Augusto O, et al. Trends in cancer incidence in Maputo, Mozambique, 1991-2008. *PLoS One* (2015) 10(6):e0130469. Epub 2015/06/26. doi: 10.1371/journal.pone.0130469. PubMed PMID: 26110774; PubMed Central PMCID: PMCPMC4481529.

22. Medina VM, Laudico A, Mirasol-Lumague MR, Brenner H, Redaniel MT. Cumulative incidence trends of selected cancer sites in a Philippine population from 1983 to 2002: A joinpoint analysis. *Br J Cancer* (2010) 102(9):1411-4. doi: 10.1038/sj.bjc.6605640.

23. Melkonian SC, Jim MA, Reilley B, Erdrich J, Berkowitz Z, Wiggins CL, et al. Incidence of primary liver cancer in American Indians and Alaska Natives, US, 1999–2009. *Cancer Causes Control* (2018) 29(9):833-44. doi: 10.1007/s10552-018-1059-3.

24. Mutyaba I, Phipps W, Krantz EM, Goldman JD, Nambooze S, Orem J, et al. A Population-Level Evaluation of the Effect of Antiretroviral Therapy on Cancer Incidence in Kyadondo County, Uganda, 1999-2008. *J Acquir Immune Defic Syndr* (2015) 69(4):481-6. Epub 2015/04/07. doi: 10.1097/qai.0000000000000620. PubMed PMID: 25844696; PubMed Central PMCID: PMCPMC4483147.

25. Njei B, Rotman Y, Ditah I, Lim JK. Emerging trends in hepatocellular carcinoma incidence and mortality. *Hepatology* (2015) 61(1):191-9. doi: 10.1002/hep.27388. PubMed PMID: 25142309; PubMed Central PMCID: PMCPMC4823645.

26. Patel N, Benipal B. Incidence of Cholangiocarcinoma in the USA from 2001 to 2015: A US Cancer Statistics Analysis of 50 States. *Cureus* (2019) 11(1):e3962. Epub 2019/04/09. doi: 10.7759/cureus.3962. PubMed PMID: 30956914; PubMed Central PMCID: PMCPMC6436669.

27. Pham C, Fong T-L, Zhang J, Liu L. Striking Racial/Ethnic Disparities in Liver Cancer Incidence Rates and Temporal Trends in California, 1988–2012. *JNCI: Journal of the National Cancer Institute* (2018) 110(11):1259-69. doi: 10.1093/jnci/djy051.

28. Pocobelli G, Cook LS, Brant R, Lee SS. Hepatocellular carcinoma incidence trends in Canada: Analysis by birth cohort and period of diagnosis. *Liver International* (2008) 28(9):1272-9. doi: 10.1111/j.1478-3231.2008.01704.x.

29. Polednak AP. Surveillance and interpretation of trends in US age-specific incidence rates for primary liver cancer, in relation to the epidemic of hepatitis C infection. *Journal of registry management* (2013) 40(3):115-21.

30. Ramirez AG, Munoz E, Holden AEC, Adeigbe RT, Suarez L. Incidence of Hepatocellular Carcinoma in Texas Latinos, 1995-2010: An update. *PLoS One* (2014) 9(6). doi: 10.1371/journal.pone.0099365.

31. Rich NE, Yopp AC, Singal AG, Murphy CC. Hepatocellular Carcinoma Incidence is Decreasing Among Younger Adults in the United States. *Clin Gastroenterol Hepatol* (2019) Accepted doi 10.1016/j.cgh.2019.04.043. doi: 10.1016/j.cgh.2019.04.043.

32. Saha SK, Zhu AX, Fuchs CS, Brooks GA. Forty-Year Trends in Cholangiocarcinoma Incidence in the U.S.: Intrahepatic Disease on the Rise. *Oncologist* (2016) 21(5):594-9. doi: 10.1634/theoncologist.2015-0446. PubMed PMID: 27000463; PubMed Central PMCID: PMCPMC4861366.

33. Shamseddine A, Saleh A, Charafeddine M, Seoud M, Mukherji D, Temraz S, et al. Cancer trends in Lebanon: A review of incidence rates for the period of 2003-2008 and projections until 2018. *Population Health Metrics* (2014) 12(1). doi: 10.1186/1478-7954-12-4.

34. Shiels MS, Engels EA, Yanik EL, McGlynn KA, Pfeiffer RM, O'Brien TR. Incidence of hepatocellular carcinoma among older Americans attributable to hepatitis C and hepatitis B: 2001 through 2013. *Cancer* (2019) 125(15):2621-30. doi: 10.1002/cncr.32129. PubMed PMID: 30980394; PubMed Central PMCID: PMCPMC6625871.

35. Shin HR, Oh JK, Lim MK, Shin A, Kong HJ, Jung KW, et al. Descriptive epidemiology of cholangiocarcinoma and clonorchiasis in Korea. *J Korean Med Sci* (2010) 25(7):1011-6. doi: 10.3346/jkms.2010.25.7.1011.

36. Siegel RL, Fedewa SA, Miller KD, Goding-Sauer A, Pinheiro PS, Martinez-Tyson D, et al. Cancer statistics for Hispanics/Latinos, 2015. *CA Cancer J Clin* (2015) 65(6):457-80. Epub 2015/09/17. doi: 10.3322/caac.21314. PubMed PMID: 26375877.

37. Sighoko D, Curado MP, Bourgeois D, Mendy M, Hainaut P, Bah E. Increase in female liver cancer in the Gambia, West Africa: evidence from 19 years of population-based cancer registration (1988-2006). *PLoS One* (2011) 6(4):e18415. Epub 2011/04/15. doi: 10.1371/journal.pone.0018415. PubMed PMID: 21490972; PubMed Central PMCID: PMCPMC3072390.

38. Song F, He M, Li H, Qian B, Wei Q, Zhang W, et al. A cancer incidence survey in Tianjin: The third largest city in China - Between 1981 and 2000. *Cancer Causes Control* (2008) 19(5):443-50. doi: 10.1007/s10552-007-9105-6.

39. Sung H, Siegel RL, Rosenberg PS, Jemal A. Emerging cancer trends among young adults in the USA: analysis of a population-based cancer registry. *Lancet Public Health* (2019) 4(3):e137-e47. doi: 10.1016/S2468-2667(18)30267-6. PubMed PMID: 30733056.

40. Tanaka H, Imai Y, Hiramatsu N, Ito Y, Imanaka K, Oshita M, et al. Declining incidence of hepatocellular carcinoma in Osaka, Japan, from 1990 to 2003. *Ann Intern Med* (2008) 148(11):820-6. PubMed PMID: 105753608. Language: English. Entry Date: 20080627. Revision Date: 20150711. Publication Type: Journal Article.

41. Thein HH, Walter SR, Gidding HF, Amin J, Law MG, George J, et al. Trends in incidence of hepatocellular carcinoma after diagnosis of hepatitis B or C infection: a population-based cohort study, 1992-2007. *J Viral Hepat* (2011) 18(7):e232-41. doi: 10.1111/j.1365-2893.2011.01440.x. PubMed PMID: 21692938.

42. Torre LA, Sauer AM, Chen MS, Jr., Kagawa-Singer M, Jemal A, Siegel RL. Cancer statistics for Asian Americans, Native Hawaiians, and Pacific Islanders, 2016: Converging incidence in males and females. *CA Cancer J Clin* (2016) 66(3):182-202. Epub 2016/01/15. doi: 10.3322/caac.21335. PubMed PMID: 26766789; PubMed Central PMCID: PMCPMC5325676.

43. Van Dyke AL, Shiels MS, Jones GS, Pfeiffer RM, Petrick JL, Beebe-Dimmer JL, et al. Biliary tract cancer incidence and trends in the United States by demographic group, 1999-2013. *Cancer* (2019) 125(9):1489-98. doi: 10.1002/cncr.31942. PubMed PMID: WOS:000465035900015.

44. Wallace MC, Preen DB, Short MW, Adams LA, Jeffrey GP. Hepatocellular carcinoma in Australia 1982 to 2014: increasing incidence and improving survival. *Liver Int* (2018). doi: 10.1111/liv.13966. PubMed PMID: 30230194.

45. Wang N, Zhu WX, Xing XM, Yang L, Li PP, You WC. Time trends of cancer incidence in urban Beijing, 1998-2007. *Chin J Cancer Res* (2011) 23(1):15-20. doi: 10.1007/s11670-011-0015-5.

46. Wang J, Li E, Yang H, Wu J, Lu HC, Yi C, et al. Combined hepatocellular-cholangiocarcinoma: a population level analysis of incidence and mortality trends. *World J Surg Oncol* (2019) 17(1):43. doi: 10.1186/s12957-019-1586-8. PubMed PMID: 30813932; PubMed Central PMCID: PMCPMC6394104.

47. Ward E, Sherman RL, Henley SJ, Jemal A, Siegel DA, Feuer EJ, et al. Annual Report to the Nation on the Status of Cancer, 1999-2015, Featuring Cancer in Men and Women ages 20-49. *J Natl Cancer Inst* (2019). doi: 10.1093/jnci/djz106. PubMed PMID: 31145458.

48. White DL, Thrift AP, Kanwal F, Davila J, El-Serag HB. Incidence of Hepatocellular Carcinoma in All 50 United States, From 2000 Through 2012. *Gastroenterology* (2017) 152(4):812-20.e5. Epub 2016/11/28. doi: 10.1053/j.gastro.2016.11.020. PubMed PMID: 27889576; PubMed Central PMCID: PMCPMC5346030.

49. Witjes CDM, Karim-Kos HE, Visser O, Van Den Akker SAW, De Vries E, Ijzermans JNM, et al. Hepatocellular carcinoma in a low-endemic area: Rising incidence and improved survival. *Eur J Gastroenterol Hepatol* (2012) 24(4):450-7. doi: 10.1097/MEG.0b013e32835030ce.

50. Witjes CD, Karim-Kos HE, Visser O, de Vries E, JN IJ, de Man RA, et al. Intrahepatic cholangiocarcinoma in a low endemic area: rising incidence and improved survival. *HPB (Oxford)* (2012) 14(11):777-81. Epub 2012/10/10. doi: 10.1111/j.1477-2574.2012.00536.x. PubMed PMID: 23043667; PubMed Central PMCID: PMCPMC3482674.

51. Xu Z, Zhou H, Lei L, Li H, Yu W, Fu Z, et al. Incidence of cancer in Shenzhen, Guangdong Province during 2001-2015: A retrospective population-based study. *Int J Environ Res Public Health* (2017) 14(10). doi: 10.3390/ijerph14101137.

52. Yeesoonsang S, McNeil E, Virani S, Bilheem S, Pittayawonganon C, Jiraphongsa C, et al. Trends in Incidence of Two Major Subtypes of Liver and Bile Duct Cancer: Hepatocellular Carcinoma and Cholangiocarcinoma in Songkhla, Southern Thailand, 1989-2030. *J Cancer Epidemiol* (2018) 2018:8267059. Epub 2019/01/25. doi: 10.1155/2018/8267059. PubMed PMID: 30675163; PubMed Central PMCID: PMCPMC6323434.

53. Zheng R, Qu C, Zhang S, Zeng H, Sun K, Gu X, et al. Liver cancer incidence and mortality in China: Temporal trends and projections to 2030. *Chin J Cancer Res* (2018) 30(6):571-9. Epub 2019/02/01. doi: 10.21147/j.issn.1000-9604.2018.06.01. PubMed PMID: 30700925; PubMed Central PMCID: PMCPMC6328503.
