# Supplementary material for: Global Trends in Incidence Rates of Primary Adult Liver Cancers: A Systematic Review and Meta-Analysis
Source: Front Oncol. 2020 Feb 28;10:171. doi: 10.3389/fonc.2020.00171 (PMC7058661; doi:10.3389/fonc.2020.00171)

Supplementary file : additional figures

Figure S4.1 Funnel plots for assessing publication bias. *Funnel plots are across included studies for overall meta-analysis of incidence trends for liver cancers combined (A), hepatocellular carcinoma (B) and intrahepatic cholangiocarcinoma (C).*

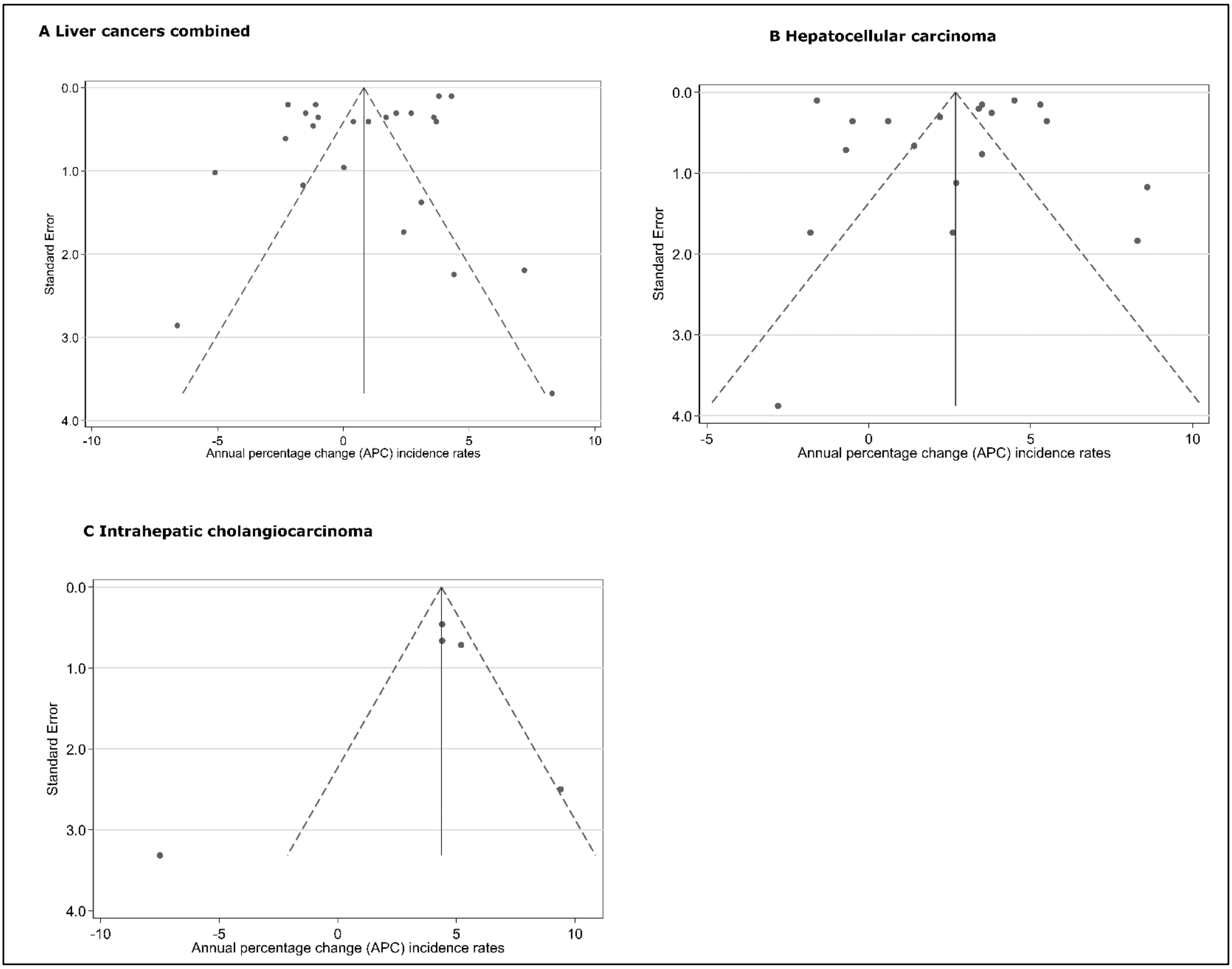

2 **Figure S4.2 Box and whiskers plot comparing studies excluded and included in meta-analysis by liver cancer type.**

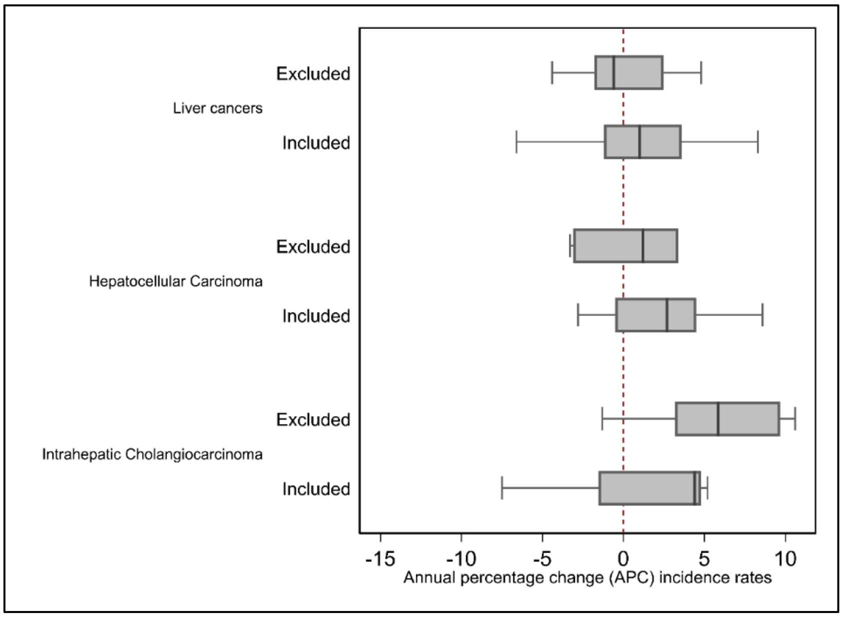

3 **Figure S4.3 Comparing excluded and included studies for meta-analysis by geographical region.**

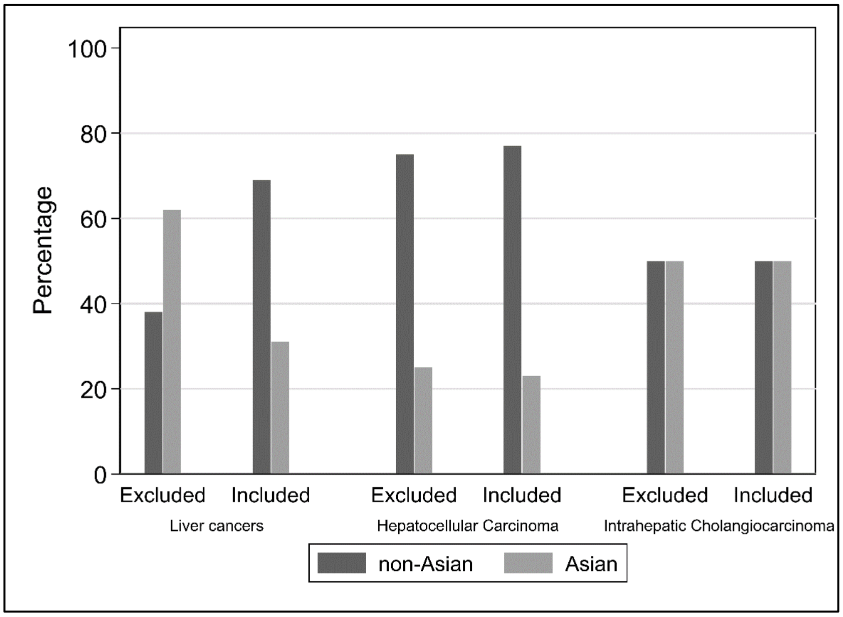

Supplement: Supplementary file 4 [file Image_1.PDF]
